# The laser pump X-ray probe system at LISA P08 PETRA III

**DOI:** 10.1107/S1600577524003400

**Published:** 2024-06-06

**Authors:** Jonas Erik Warias, Lukas Petersdorf, Svenja Carolin Hövelmann, Rajendra Prasad Giri, Christoph Lemke, Sven Festersen, Matthias Greve, Philippe Mandin, Damien LeBideau, Florian Bertram, Olaf Magnus Magnussen, Bridget Mary Murphy

**Affiliations:** a Institute of Experimental and Applied Physics Kiel University Leibnizstrasse 19 24118Kiel Germany; b Ruprecht-Haensel Laboratory, Olshausenstrasse 40, 24098Kiel, Germany; c Deutsches Elektronen-Synchrotron DESY Notke­strasse 85 22607Hamburg Germany; d Universite de Bretagne Sud IRDL, UMR CNRS 6027 Lorient France; NSRRC, Taiwan

**Keywords:** pump–probe instrumentation, liquid interfaces, optical excitation, structural dynamics, X-ray scattering

## Abstract

Understanding and controlling the structure and function of liquid interfaces is a constant challenge in biology, nanoscience and nanotechnology, with applications ranging from molecular electronics to controlled drug release. X-ray reflectivity and grazing incidence diffraction provide invaluable probes for studying the atomic scale structure at liquid–air interfaces. The new time-resolved laser system at the LISA liquid diffractometer situated at beamline P08 at the PETRA III synchrotron radiation source in Hamburg provides a laser pump with X-ray probe. The femtosecond laser combined with the LISA diffractometer allows unique opportunities to investigate photo-induced structural changes at liquid interfaces on the pico- and nanosecond time scales with pump–probe techniques. A time resolution of 38 ps has been achieved and verified with Bi. First experiments include laser-induced effects on salt solutions and liquid mercury surfaces with static and varied time scales measurements showing the proof of concept for investigations at liquid surfaces.

## Abstract 2

Pump–probe experiments are possible on liquid interfaces using the LISA instrument at P08 beamline PETRA III, DESY. The time scales available range from 38 ps to seconds.

## 1. Introduction

In nature, liquid interfaces are widespread and play a key role in diverse processes including biological function, chemical processing and environmental science (Schlossman, 2002 ▸; Jonge & Ross, 2011 ▸; Hyman *et al.*, 2014 ▸; Eisenthal, 1996 ▸). Understanding and controlling the structure and function of liquid interfaces are constant challenges in environment science, biology and nanoscience with applications ranging from solar thermal cells to biological membranes. Despite intensive research, many aspects are still open. The local order and dynamics of liquids undergo significant changes near liquid interfaces, leading to a variety of phenomena including changes in charge distribution and chemical composition close to the interface (Miller *et al.*, 1995 ▸) and can result in atomic or molecular layering (Elsen *et al.*, 2010 ▸; Luo *et al.*, 2013 ▸). Computer simulations (Kessler *et al.*, 2015 ▸) and experimental investigations with optical spectroscopy methods (Miranda & Shen, 1999 ▸; Ni & Skinner, 2015 ▸) and electron spectroscopy (Benjamin, 2006 ▸) have also shown that the structure and dynamics of the fluid in the near-interface region deviate significantly from the collective behaviour in the bulk. These studies contribute to understanding important natural and technological processes, *e.g.* electron transfer, heterogeneous catalytic reactions and biomolecular processes.

While spectroscopic studies of ultrafast dynamics at liquid interfaces (Tamarat *et al.*, 2000 ▸) provide insight into the behaviour of individual molecules such as vibrations, rotations or electronic states, X-ray scattering techniques provide an invaluable probe to determine the interface structure (Pershan & Schlossman, 2012 ▸). In particular, X-ray reflectivity (XRR) and grazing incidence diffraction (GID) are central to understanding the interfacial structure at liquid interfaces (Haddad *et al.*, 2018 ▸) including layering of metallic liquids (Pershan & Schlossman, 2012 ▸; Elsen *et al.*, 2013 ▸) and the structural phase behaviour of organic Langmuir films on liquid sub-phases (Kraack *et al.*, 2003 ▸; Stefaniu *et al.*, 2014 ▸). Among other methods, they also provided insight into the equilibrium dynamics of liquid surfaces, including capillary wave behaviour (Runge *et al.*, 2016 ▸). Clear deviations from the classical capillary wave theory on short length scales observed in various studies (Fradin *et al.*, 2000 ▸; Elsen *et al.*, 2010 ▸) can be partially explained by molecular dynamic simulations (Sega & Dellago, 2017 ▸) and are still the subject of current research (Nguyen & Tran, 2018 ▸; Seo *et al.*, 2018 ▸; Zhang *et al.*, 2021*a* ▸). Pump–probe studies of bulk water following the high-to-low density transition in amorphous ice could determine pico­second dynamics separating the anisotropic scattering decay with a time scale of 160 fs from the delayed temperature increase on the picosecond time scale (Perakis *et al.*, 2017 ▸; Ladd-Parada *et al.*, 2022 ▸). Simulations and spectroscopic studies of liquid interfaces and liquid jets indicate that the dynamics for orientation-relaxation, diffusion, solvation and intermolecular energy transfer are influenced by the presence of the interface (Jungwirth & Tobias, 2006 ▸; Petersen & Saykally, 2006 ▸; Dallari *et al.*, 2021 ▸; Fu *et al.*, 2009 ▸; Biasin *et al.*, 2021 ▸; Kim *et al.*, 2020 ▸; Onufriev & Izadi, 2018 ▸).

Highly topical ultrafast dynamic studies by pump–probe X-ray scattering on solids such as spin-crossover crystals (Collet *et al.*, 2012 ▸), gas phase molecules (Yong *et al.*, 2021 ▸) and bulk liquids such as solvated Co(II) compounds (Biasin *et al.*, 2016 ▸) or molecular-level water dynamics (Kim *et al.*, 2020 ▸) demonstrating femtosecond to picosecond responses are already well established. A number of beamlines at synchrotrons allow pump–probe measurements but focus on solid samples or transmission in liquid geometries, for example at the European Synchrotron Radiation Source (Wulff *et al.*, 2003 ▸), SPring-8 (Fukuyama *et al.*, 2008 ▸), the Advanced Photon Source (March *et al.*, 2011 ▸), BESSY II (Navirian *et al.*, 2012 ▸), Soleil (Laulhé *et al.*, 2013 ▸), KIT Light Source (Issenmann *et al.*, 2013 ▸), Beijing Synchrotron Radiation Facility (Wang *et al.*, 2017 ▸), MAX IV Laboratory (Enquist *et al.*, 2018 ▸), Elettra-Sincrotrone Trieste (Burian *et al.*, 2020 ▸) and Stanford Synchrotron Radiation Lightsource (Reinhard *et al.*, 2023 ▸), among others. There are also a large number of setups at free-electron laser facilities focusing on similar topics on even faster time scales.

However, analogous studies at liquid surfaces and interfaces have not yet been reported despite increasing interest. Ultrafast surface-sensitive X-ray scattering experiments could provide new data relevant to understanding the dynamics of these interfaces, for example on the redistribution of the capillary wave spectrum upon thermal excitation or on the relaxation of local interfacial order at nanosecond or pico­second time scales. Such measurements could also contribute to the understanding of the non-equilibrium dynamics of Langmuir films, which have been rarely studied so far (Hobley *et al.*, 2008 ▸; Chandran *et al.*, 2015 ▸). However, such studies are highly demanding and pose new challenges, as compared with established pump–probe studies of bulk materials or solid surface. In particular, fluidic surfaces may be deformed or mechanically excited under laser irradiation, specifically for irradiation by high power pulses. While true time-resolved measurements on the picosecond time scale are the holy grail of such studies, even time-averaged data obtained by conventional surface X-ray scattering may provide interesting insights into the effects of pulsed laser excitation, as illustrated recently in studies of the laser annealing of metallic glasses (Antonowicz *et al.*, 2021 ▸).

Here we describe first steps towards the implementation of time-resolved laser excitation studies at fluidic interfaces. We first report on instrumental developments at the Liquid Interface Scattering Apparatus (LISA) (Murphy *et al.*, 2014 ▸) at beamline P08 (Seeck *et al.*, 2012 ▸) of the PETRA III synchrotron source, which provides a useful platform for investigating structural properties at liquid surfaces. The addition of an optical-pump/X-ray-probe facility at P08, described in this paper, allows experiments on laser-induced phenomena at liquid interfaces at LISA and in other systems at the six-circle Kohzu diffractometer. We then provide results of first experiments on salt solutions and liquid metals by this setup, focusing on the ‘static’, *i.e.* time-averaged, scattering signals observed during laser irradiation.

## 2. LISA at P08

The high-resolution beamline P08 at PETRA III (Seeck *et al.*, 2012 ▸) offers a wide range of possible experimental setups suitable for experiments on liquid–liquid and liquid–vapour interfaces at the LISA diffractometer (Pattadar *et al.*, 2021 ▸; Haddad *et al.*, 2018 ▸; Sartori *et al.*, 2022 ▸) or the Langmuir Grazing Incidence Diffraction setup (Shen *et al.*, 2022 ▸) and experiments at the multifunctional six-circle diffractometer from Kohzu Precision, Kawasaki, Japan (Fadaly *et al.*, 2020 ▸; Zhang *et al.*, 2021*b* ▸; AlHassan *et al.*, 2020 ▸). The beamline is situated on the high-beta section at the PETRA III third-generation synchrotron radiation source providing a highly monochromatic low divergent X-ray beam. The X-ray beam, delivered by an undulator followed by a double crystal and a large offset monochromator, has photon energies ranging from 5.4 to 29.4 keV with an energy resolution between Δ*E*/*E* = 8 × 10^−6^ and 6 × 10^−5^. The beamline offers various focusing options resulting in different beam sizes, energy resolution and beam divergence. A detailed description has been given by Seeck *et al.* (2012 ▸). For typical LISA operation, the beam is moderately focused resulting in a beam size of 300 µm × 500 µm with a divergence of 11 µrad × 20 µrad at 18 keV. Furthermore, slits are used to cut down the beam size to 100 µm × 400 µm.

PETRA III provides X-ray bunches with a repetition rate of 5.2 MHz and a beam current dependent bunch length of ∼100 ps full width at half-maximum (FWHM) (Balewski *et al.*, 2011 ▸) in the 40-bunch mode also known as the timing mode. This results in a gap of 192 ns between each bunch.

**Figure 1:**
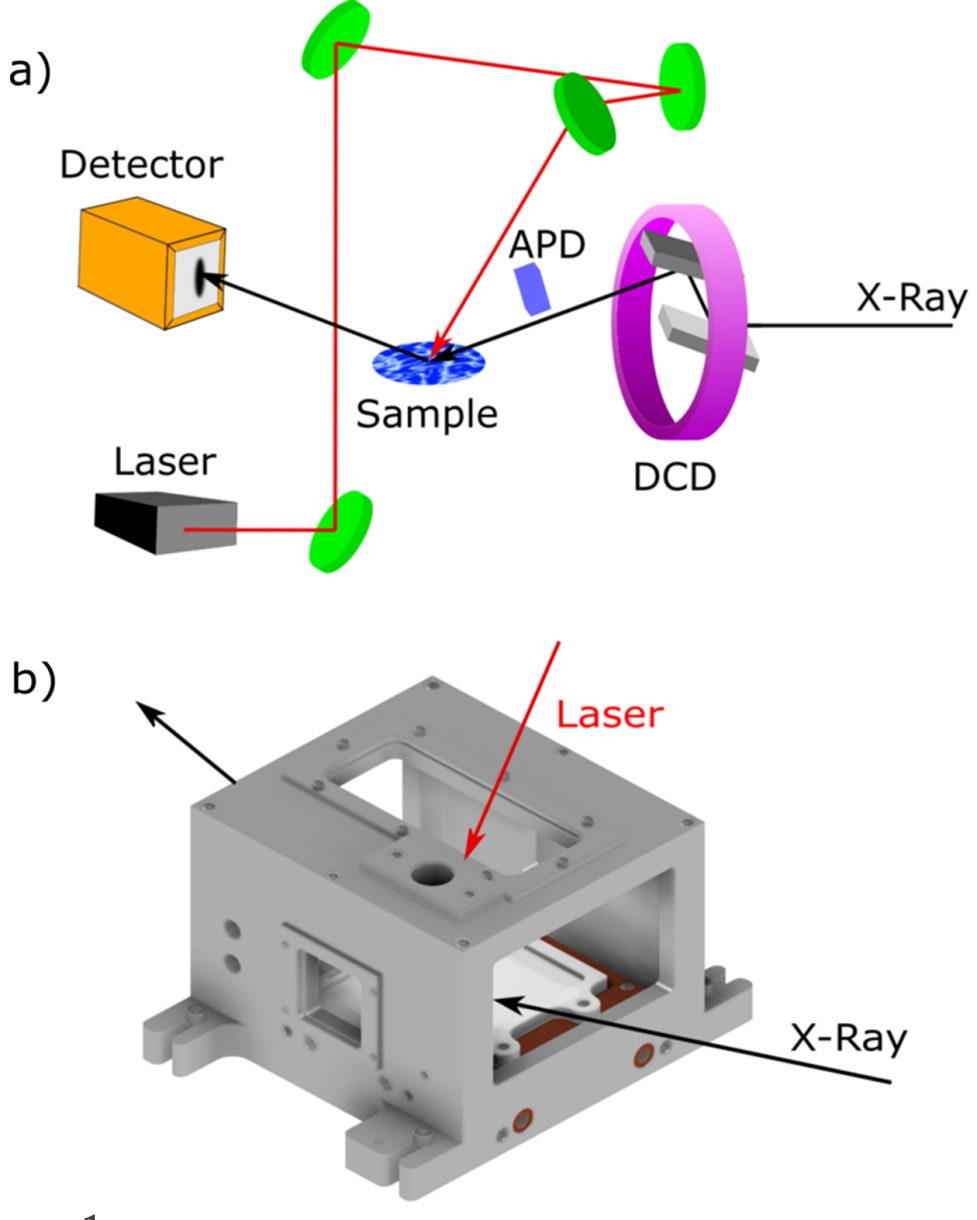
Schematic of the LISA and laser setup installed at P08. (*a*) The laser and X-ray beam paths at the LISA instrument via the double crystal deflector (DCD) is indicated in red and the X-ray in black. (*b*) Schematic drawing of the liquid cell used at the beamline.

LISA operates in reflection geometry and tilts the X-ray beam onto the sample without requiring the sample to be moved which is convenient for investigating liquid interfaces (Fig. 1 ▸). LISA consists of three vibration decoupled elements: the double crystal deflector (DCD), the sample stage and the detector stage. The DCD consists of Ge (111) and Ge (220) crystals. By rotating the pair of asymmetric crystals around the incoming X-ray beam axis the angle of incidence is changed without sample movement. The angular resolution for all subcomponents is a minimum of one order of magnitude below the PETRA III beam divergence, which therefore determines the resolution together with the detector setup. For the timing measurements, we used an X-spectrum Lambda 750k GaAs detector with 55 µm pixel size at a distance of up to 1.2 m from the sample. These detectors allow gated data acquisition with respect to the PETRA III bunch clock (see Section 3.1) and thus enable ultrafast scattering experiments. In addition, both single image and ‘burst mode’ measurements, *i.e.* a fast series of images, are available for single and multiple pulse laser shots.

The diffractometer and its components are operated by the control system Sardana/TANGO. The detector images are stored in NeXus file format along with other relevant beamline data in a separate ascii file in the storage system.

## 3. Laser setup and parameters

**Figure 2:**
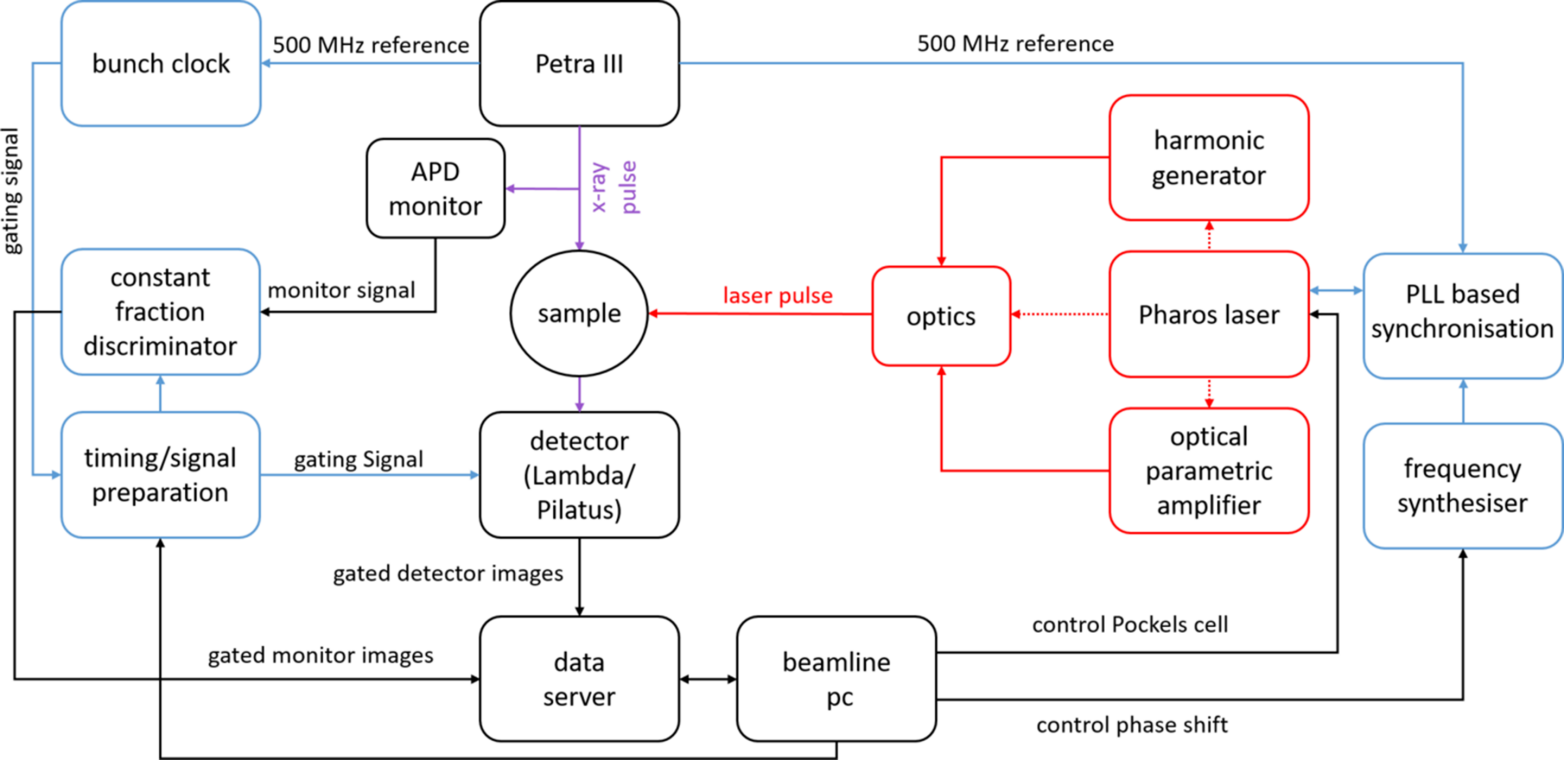
Schematic of the laser-pump/X-ray-probe setup at P08 PETRA III. The laser system is synchronized and shifted in time via a phase locked loop (PLL) with the PETRA III master clock. By using gated detectors (Lambda, Pilatus) and monitors (APD-avalanche photodiode) gated with the PETRA III bunch clock, the probed X-ray pulse can be selected and measured. Laser components are illustrated in red and timing components in blue.

Within the work described here, LISA was equipped with a femtosecond pulsed laser system in a separate laser hutch below the P08 control hutch. A combination of manual and motor-controlled mirrors guides the laser beam from the laser hutch to the sample (Fig. 1 ▸). A block diagram of the pump–probe setup is shown in Fig. 2 ▸. The laser system consists of a Pharos femtosecond laser from Light Conversion with a fundamental wavelength of 1030 nm and maximum output power of 15 W. The laser pulse length ranges from 251 fs up to 12 ps with frequency ranging from 100 kHz up to 1041 kHz. This results in pulse energies from 14 µJ to 150 µJ. With an externally controllable Pockels cell at the output of the laser unit, it is also possible to remotely control the repetition rate of the final output to produce integer subharmonics of the maximum set frequency or reduce the pulse energy further.

The Pharos laser may be used directly to deliver a wavelength of 1030 nm or to pump one of the additional Light Conversion modules, namely the higher harmonic generator known as the Hiro module or the optical parametric amplifier Orpheus to provide alternative wavelengths. In our setup, the Hiro module is optimized to generate the second, third and fourth harmonic with optimized efficiency with input frequency of 130 kHz and pulse length of 251 fs. The Orpheus can generate a wide wavelength range from 210 to 2600 nm at 1041 kHz and 251 fs but provides a lower power. Typical output powers are listed in the supporting information. Motorized wavelength-dependent mirror stages direct the beam to the LISA experimental station, as indicated in Fig. 1 ▸, via a shielded laser path with a total length of up to 10 m. The laser beam impinges on the sample from above at an angle of 30° with respect to the axis normal to the sample surface in order to free the space directly above the sample cell, which is often used for a micro-focus camera system to monitor the sample state. Nevertheless, an option for hitting the sample perpendicular to its surface with the laser beam also exists. The linear polarized laser light can be shifted either horizontally or vertically. Breadboards along the laser beam path are installed at every mirror box to allow the installation of additional equipment including lenses, beam shaping devices *etc*. Additionally, there is a laser path to the Kohzu diffractometer also present in the P08 experimental hutch, making the laser also available for user experiments at this diffractometer.

### 3.1. Synchronization

The laser oscillator is synchronized to a single bunch at the PETRA III synchrotron via phase-locked-loop (PLL) electronics as shown in Fig. 2 ▸. RRE-Synchro Repetition Rate Stabilization electronics (Menlo Systems, 2014 ▸) are used to phase lock the laser oscillator to the sixth subharmonic of the 499.6655 MHz RF PETRA-III master clock (Klute *et al.*, 2011 ▸) signal (further referred to as 500 MHz in this article). A Menlo Systems DDS120 direct digital synthesizer (Menlo Systems, 2009 ▸) acts as a phase shifter to realize a relative time shift between the laser pulse and the selected X-ray bunch. The timing electronics was purchased together with the laser system. The details of synchronization electronics, PLL and DDS120 are described elsewhere (Schlie, 2013 ▸; Menlo Systems, 2009 ▸, 2014 ▸). As outlined in the references above, in our setup the 500 MHz PETRA III signal is frequency doubled and mixed with a 20 MHz low noise reference to provide a band-pass filtered 980 MHz signal which serves as a reference for the phase distortion (PD) unit. The oscillator repetition rate is detected with an optical fibre coupled photon detector providing a 1 GHz signal. This 1 GHz signal is mixed and band-pass filtered with the 980 MHz reference resulting in a 20 MHz signal with a specific phase error. By mixing the resulting 20 MHz signal with a phase adjustable 20 MHz signal from the DDS120 synthesizer the final overall phase error is generated. The Pharos laser cavity length is adapted to correct the phase error via two piezoelectric actuators driven by the RRE-Synchro. By adjusting the phase of the DDS120 synthesizer the laser can be phase shifted with respect to the master clock and hence to the X-ray pulse. According to the manufacturer specification, the minimum attainable phase shift is 0.05° corresponding to 142 fs. Using this approach, a time delay ranging from −10 ns to +10 ns, with a resolution of 142 fs could be achieved.

**Figure 3:**
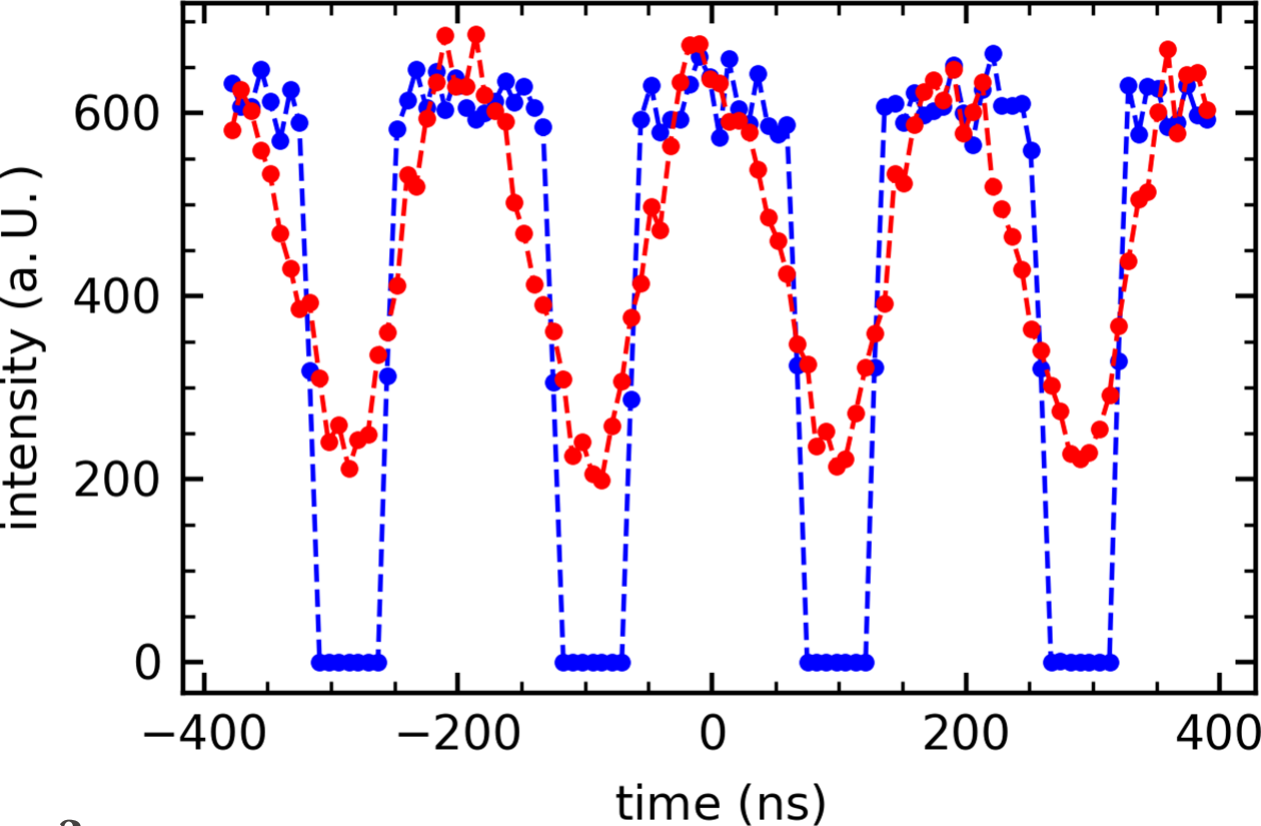
The bunch clock pattern as measured by the GaAs Lambda Detector (red) and the APD (blue) measured at LISA for a 130 kHz gating signal with a 128 ns width. PETRA III was operating in the 40 bunch timing mode.

Our setup allows the laser to be operated at different frequencies which are the subharmonics of the PETRA III frequency. An electronically gated detector after the sample and multiple up line monitors allow extraction of the chosen X-ray bunch or multiple X-ray bunches associated with each laser pulse depending on the detector gate width and frequency. This facilitates variation of the X-ray pulse frequency and width. The X-spectrum Lambda 750k has been implemented as the gated detector. The detector gating signal is provided by the PETRA III bunch clock and is generated from the 500 MHz RF signal. Depending on the frequency of the laser, the gating signal is provided by an appropriate raster from the bunch clock. The gating signal is then prepared individually for each detector and monitor. The gating signal is also integrated into the beamline control system and measurement timing, before providing it to the detector. An avalanche photodiode (APD) from FMB Oxford Instruments operated in gating mode is used as a monitor for the normalization of the time-resolved data. The SCA output signal from APD ACE electronics (FMB-Oxford) is processed together with the gating signal in a constant fraction discriminator (Ortec 939 QUAD CFD) to extract the gated intensity of the X-ray bunch. Fig. 3 ▸ shows the PETRA III bunch structure measured with different detectors in the 40 bunch mode. In blue, the gated signal of the APD is shown, while the red signal shows the Lambda detector. Both detectors were gated to a 130 kHz bunch clock signal with a gate width of 128 ns, selecting one X-ray bunch from 40. By shifting the bunch clock signal the X-ray bunches were rastered to check the bunch structure and to ensure that a single X-ray bunch is selected at the centre of the gating window. For the attained jitter for the signal measured by the Lambda of roughly 50 ns, centring on the selected bunch ensures that only the chosen bunch is detected and reduces the signal contribution from neighbouring bunches to a negligible level. The X-ray bunches could be resolved nicely by the gated APD with a delay of fewer than 16 ns between high (gated X-ray bunch) and low (gating between two bunches). Unlike the APD signal, the measured minimum signal never reaches 0 counts for the Lambda as the time resolution is less well resolved. However, the centres of maxima and minima match well with those of the APD, confirming the timing overlap between the Lambda and APD. It was not possible to further shorten the gate width as otherwise the full intensity of the bunch could not be measured. Using this method, a maximum time delay of 80 ps is achievable.

By jumping between the X-ray bunches, which are separated by 192 ns, it was possible to extend the time delay and hence the available time range up to 7.68 µs. The latter corresponds to the periodicity of all 40 bunches in the ring with 192 ns time separation and is achieved by shifting the bunch clock signal and thus the detector gating in time. Obviously, the time resolution in this mode is fixed at 192 ns. A time delay of microseconds to milliseconds was achieved by controlling the Pharos Pockels cell with an additional synchronized electronics.

### 3.2. Spatial and temporal overlap

**Figure 4:**
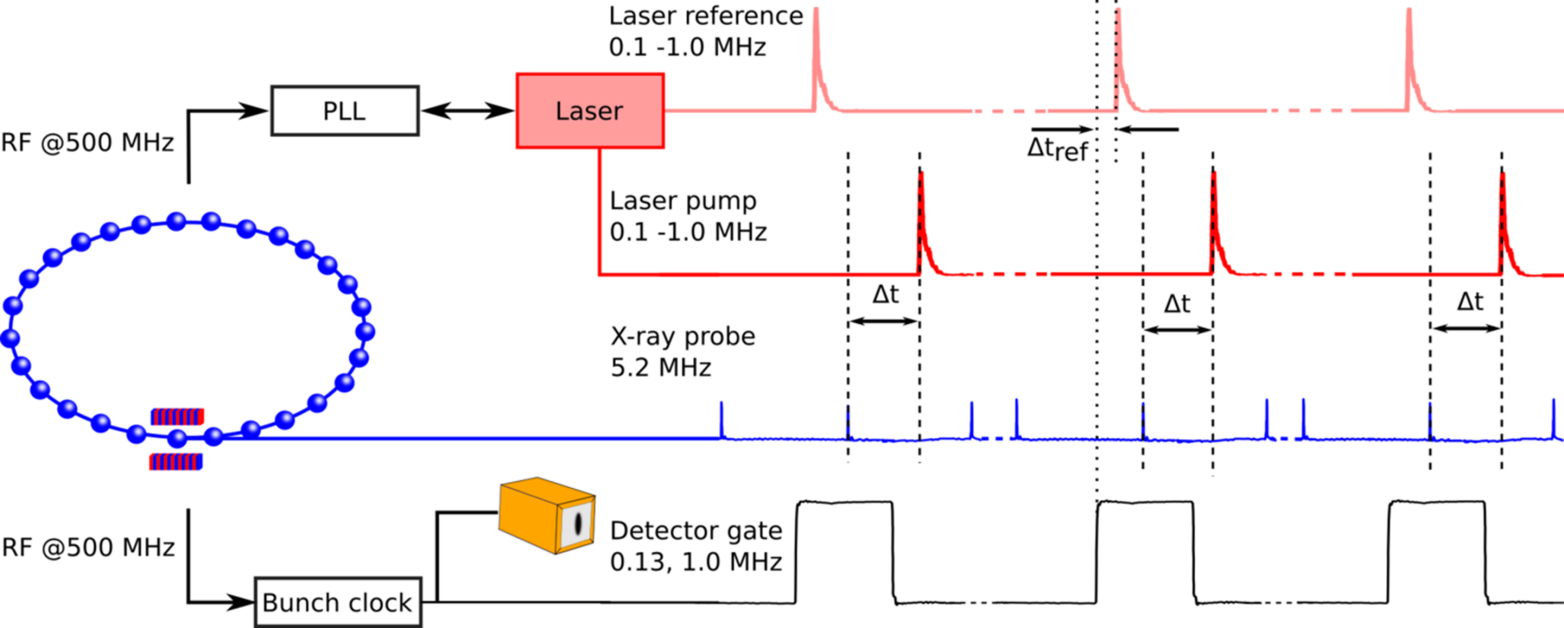
An oscilloscope trace showing the detector gate signal generated from the bunch clock (black), the laser reference signal (light red) produced by one of the photodiodes in the Pharos laser unit, the X-ray bunch (blue) and laser signal (red) from the photodiode at the sample position.

The spatial overlap of the laser and X-ray beam spots at the sample position is confirmed by a photodiode. A fast photodiode (AXUVHS6; Opto Diode Corp.) with an active area of 0.0008 mm^2^ and a rise time of 50 ps is connected via a 100 kΩ resistor and an amplifier (Mini-Circuits, ZFL-2500VH+) to an oscilloscope (Waverunner 640Zi) with a maximum sample rate of 40 GS s^−1^. The diode is placed in the X-ray beam at the sample position directly at the pivot point of the LISA diffractometer. The diode is sensitive to both the X-rays and laser signals. Once the X-ray beam is detected on the diode, the laser beam is moved via the motorized mirror before the sample to maximize the laser signal on the oscilloscope. This confirms the spatial overlap. The temporal overlap between the signals is illustrated in Fig. 4 ▸, which shows such oscilloscope data during this procedure. The light red signal shows a reference signal from a photodiode directly at the laser output. The black line is the detector gating signal received from the PETRA III bunch clock. It allows separation of the individual X-ray bunches. The blue line indicates the photodiode signal of the X-ray bunches at the sample position. The red signal originates from the laser signal at the sample position. To achieve temporal overlap the laser pulse is shifted via the laser phase shifter until the laser and X-ray signals overlap on the oscilloscope (Δ*t* = 0). Depending on the laser pulse parameter this method allows the pulse positions to be matched with an accuracy of 10 ps to 50 ps. A more precise adjustment is achieved by measuring the laser-induced thermal expansion along a lattice plane of a bis­muth (111) thin film sample with a typical response on a time scale of picoseconds, as described later. After temporal alignment of the pulses the time delay between the triggered edge of the bunch clock signal and the laser reference (Δ*t*_ref_, shown in Fig. 4 ▸) coming from one of the photodiodes in the Pharos laser unit is noted. This value is observed throughout the experiment as it is considered to be a confirmation of the timing overlap. Since the bunch clock is fed by the PETRA III RF signal, any possible relative temporal shift of the X-ray pulse caused by any unforeseen reasons such as beam dump also affects the bunch clock signal and results in a deviation of the time delay from the noted value. In addition, deviations can also originate from the temporal movement of the laser (caused by, for example, a jump introduced by the timing electronics). For all of these reasons, occasional realignment of the temporal overlap is deemed necessary if Δ*t*_ref_ changes at Δ*t* = 0.

## 4. Picosecond temporal resolution determined by bismuth lattice response

**Figure 5:**
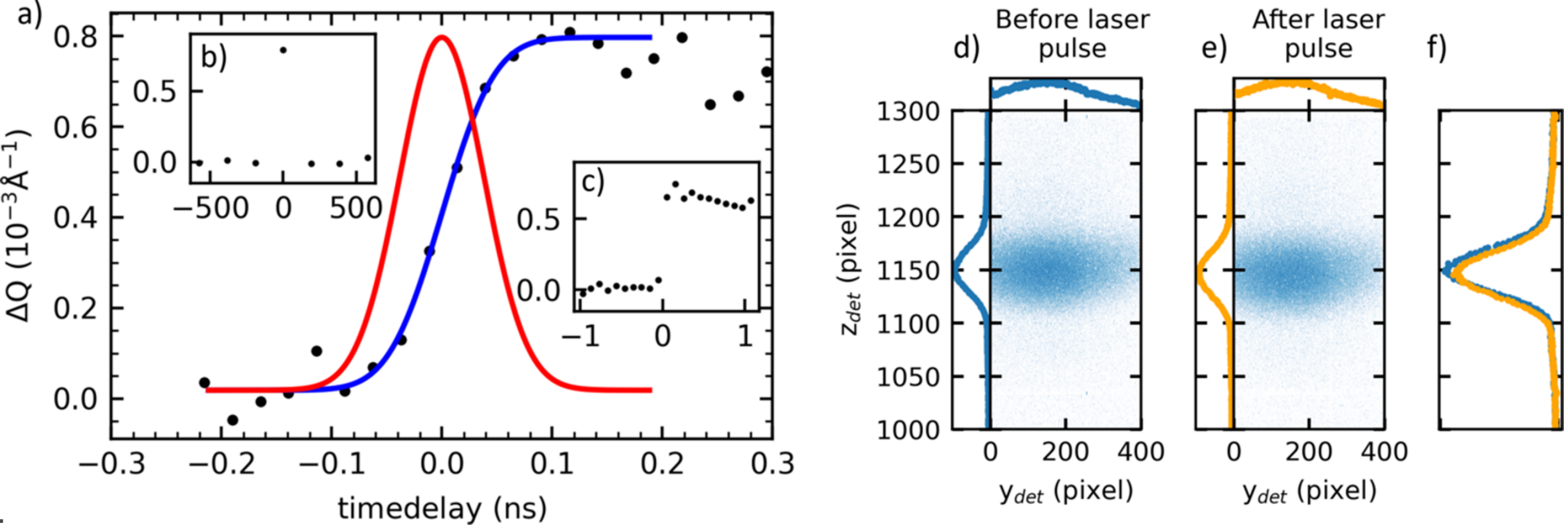
(*a*) Bi (111) Bragg peak shift (black dots) and fit (blue line). The red curve is the derivative (in arbitrary units) of the fit. Inset (*b*) shows the response on a 1000 ns and (*c*) on a 2 ns time scales. The bis­muth sample was excited by a 1030 nm, 12 ps laser pulse with a fluence of 182.4 µJ cm^−2^. Raw detector images are shown in (*d*) before and (*e*) after the laser pulse with the integrated intensity profiles before (blue) and after (orange) shown in (*f*).

To benchmark the setup, characterize the temporal resolution and precisely check the temporal overlap, the thermal lattice expansion of the bis­muth thin film was measured. This is reported to occur in the picosecond time scale (Laulhé *et al.*, 2013 ▸; Esmail *et al.*, 2013 ▸; Bugayev & Elsayed-Ali, 2019 ▸). A (111) orientated bis­muth layer with a thickness of about 80 nm was prepared by evaporation on an Si(001) wafer. The thermal expansion of the Bi (111) Bragg peak following the excitation by a single laser pulse was measured using the pump–probe setup. A 1030 nm laser pulse with 12 ps (FWHM) pulse length, 31.2 µJ pulse energy and a diameter of 6.6 mm (1/e^2^) resulting in a fluence of 182.4 µJ cm^−2^ was chosen as the optical pump, followed by an 18 keV X-ray pulse as the probe, with variable time delay Δ*t* between the laser and the X-ray pulses. Fig. 5 ▸ shows the relative peak position of the Bi (111) peak, obtained by fitting the two-dimensional detector images with a Gaussian profile, for a range of time delays between pump and probe pulses. Two example detector images before and after a laser pulse are shown in Fig. 5(*b*) ▸. For this experiment the temporal shift of the synchronized laser pulse was achieved by shifting the phase-locked loop as described above. A clear shift of 0.8 × 10^−3^ Å^−1^ of the bis­muth (111) Bragg peak is observed. The standard deviation was determined to be σ_total_ = 38 ± 6 ps by a fit of a cumulative distribution function (blue line) to the measured data (black dots) and represents the total time resolution of the setup. The resolution is determined by the time of lattice expansion of the bis­muth sample, the pulse length of the X-ray and laser pulse, as well by the jitter between them introduced by the synchronization electronics. Assuming a Gaussian shaped laser and X-ray pulses and a Gaussian jitter contribution, the resulting convolution has been assumed as a Gaussian. Thus, the standard deviations are the sum of the squares. Since the length of the laser pulse σ_laser_ = 5.1 ps and the jitter of the synchronization electronics σ_jitter_ ≤ 2 ps only contribute in the sub-picosecond regime, the resolution is primarily determined by the X-ray pulse length and the time of the lattice expansion. To estimate the time scale of the bis­muth lattice expansion we have evaluated previously published data. A similar experiment (Laulhé *et al.*, 2013 ▸) reports an initial shift of the Bragg peak below 80 ps, which we estimated to be σ_lattice_ < 17 ps from their data. Other data taken by electron diffraction methods (Esmail *et al.*, 2013 ▸; Bugayev & Elsayed-Ali, 2019 ▸) report even shorter time scales with σ_lattice_ < 6 ps. In all cases the contribution to our resolution is below 3 ps and within our errors. This suggests that the X-ray pulse length is the main factor limiting our resolution. The measured resolution of σ_total_ = 38 ± 6 ps is in accordance with the PETRA III pulse length design value of 40 ps (Balewski *et al.*, 2011 ▸) and is slightly shorter than previous measured values of 47 ps for the used bunch energy of 2.5 mA (Balewski *et al.*, 2011 ▸). The inset of Fig. 5 ▸(*a*) shows the measurement over a wide range of time delays, covering a range of 1 µs, by scanning the X-ray bunches around the bunch overlapping with the laser pulse as described above. In this case, the gating signal coming from the bunch clock was delayed in steps of ±192 ns to move to the adjacent bunches. Also, an additional time delay of ∼30 ps was introduced between the laser and X-ray pulses during this scan in order to achieve a visible thermal effect at the X-ray bunch considered as time zero. The lattice expansion was observed to return to 0 already in the X-ray bunch next to the time zero bunch, indicating a decay of the thermal expansion within 192 ns. This procedure further ensures that the correct bunch, which is associated with a laser pulse, is selected out of the 40 bunches in the adjustment of the gating setup.

## 5. First static measurements and laser influence on liquid surfaces

To illustrate the performance and potential science cases for the new laser-pump/X-ray-robe setup on liquid–vapour interfaces we performed first prototype experiments on liquids. In liquid salt solutions the specific ion species can cause very different phenomena despite similar electron structure, such as Cl^−^ or Br^−^. In the field of atmospheric physics (Rhew *et al.*, 2000 ▸; Oum *et al.*, 1998 ▸; Nepolian *et al.*, 2021 ▸), and also in other areas of technical applications (Chin *et al.*, 2001 ▸; Meng *et al.*, 2022 ▸), exposure of salt solutions to radiation plays an important role. The type and energy of radiation have different influences on their properties. Specifically, UV radiation plays a major role here, as it is emitted by the sun and absorbed in the atmosphere, as well as often used in technical processes. Previous studies showed that UV irradiation can excite photoelectrons in water and salt solutions, resulting in solvated electrons (Sauer *et al.*, 2004 ▸). Therefore, first experiments on liquid surfaces were carried out at the interface of a water-based salt solution and vapour, using a 258 nm UV laser for the electronic excitation and X-ray reflectivity (XRR) to investigate the influence of laser pulse irradiation on the liquid interface structure. In addition, experiments were performed from a mercury–vapour interface to study heat-induced structural changes induced by the laser at 1030 nm. These measurements aimed at obtaining preliminary data for future ultrafast studies of the non-equilibrium dynamics of solvated electrons and capillary waves at liquid interfaces using the XRR technique.

The cell used for the studies of water and aqueous salt solutions consists of a water-cooled Teflon Kel-F trough of dimensions 40 mm × 90 mm surrounded by an aluminium housing to achieve a controlled N_2_ atmosphere. The cell has a quartz glass plate of 1 mm thickness on the top and two X-ray Kapton windows to allow simultaneous illumination by laser and X-rays as illustrated in Fig. 1 ▸(*b*). The thin bottom (0.5 mm) and low height (2 mm) of the trough ensures a good heat transport to the copper base plate, which can be cooled by a JULABO chiller and/or an additional Peltier. A schematic drawing of the liquid cell is shown in Fig. 1 ▸(*b*) and in Fig. S3 of the supporting information. The cell used for the mercury experiments was an ultra-high vacuum compatible stainless steel (SS) chamber and is shown in Fig. S2. The cell consists of an inner cell with a diameter of 50 mm and a depth of 5 mm and a quartz window at the top for the laser. The laser window also serves as a viewport for an optical microscope (Basler ACE acA1600-20gm with OPTEM Fusion motorized Zoom system 12.5:1) to monitor the sample state. A mercury reservoir, viewport, scraper, gas inlet and outlet and turbomolecular pump equipped with a vacuum gauge are connected to the cell through the high vacuum ports. The cylindrical reservoir made of SS contains approximately 20 ml of mercury and is connected via a SS rod extended up to the inner cell. The mercury surface has been tested to remain flat over more than 25 mm at the centre which is sufficient for X-ray reflectivity measurements. The scraper made of an Mo plate is mounted on a rotatable bellow to remove any unwanted oxide layer deposited on the mercury surface. To reduce the oxygen partial pressure, the cell is evacuated and backfilled with an N_2_–H_2_ (96:4) mixture for three cycles and finally filled in with H_2_ gas. A small flow of H_2_ is also maintained throughout the experiment to keep the sample clean for a longer period. The cell is operated in the temperature range between −2 and 3°C by water flow using a JULABO chiller connected to the base plate of the inner cell. Mercury (99.999+%) and NaI (99.99%) were purchased from Sigma Aldrich. The salt was baked for more than 12 h at temperatures above 300°C to eliminate any possible organic contamination and moistures. The solutions of different salt concentrations were prepared with 18 MΩ Milli-Q water (Elga Purelab Ultra Analytic, 18 MΩ cm^−1^).

### 5.1. Laser excitation of NaI solution

**Figure 6:**
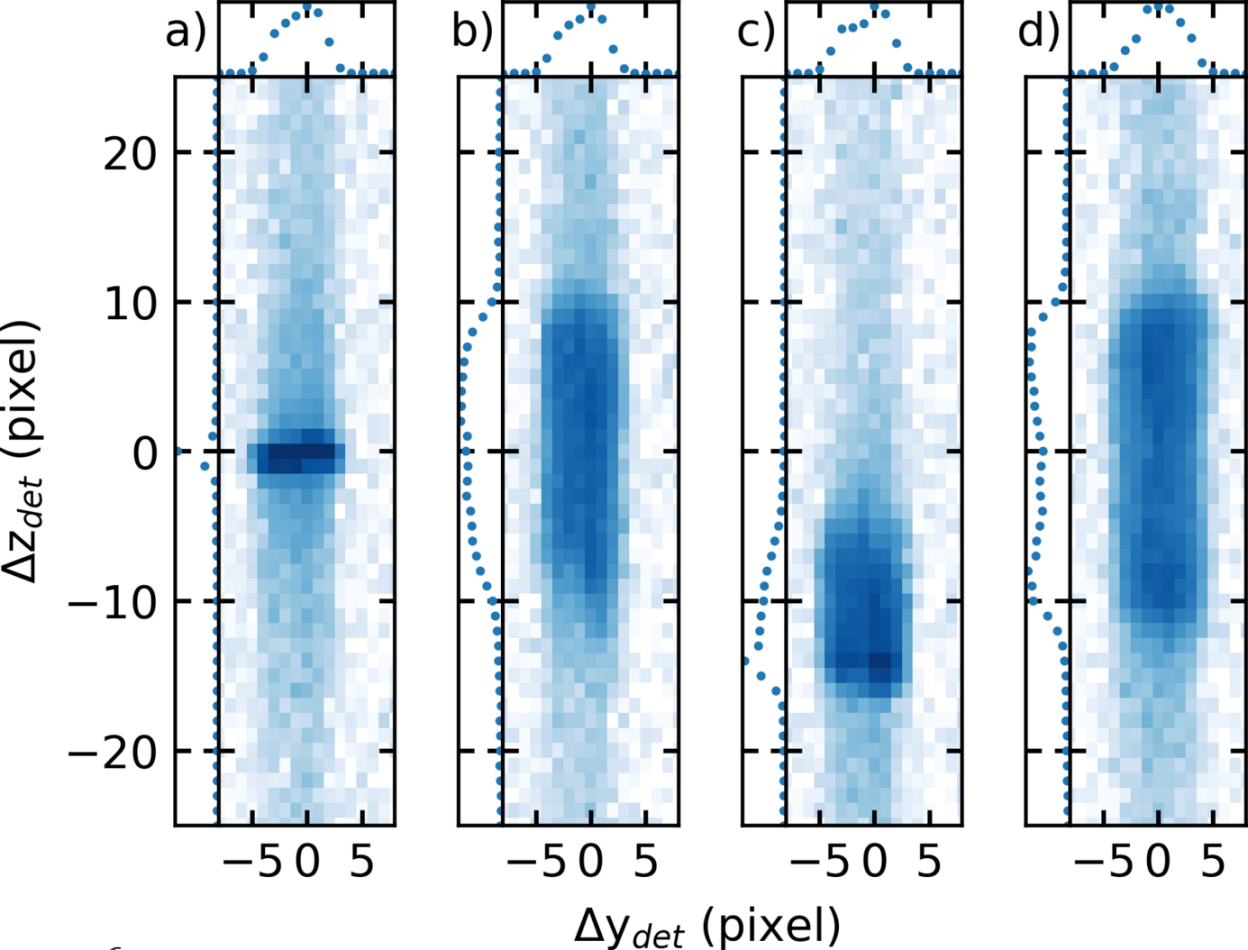
Detector images of the specular reflected beam from a 0.5 *M* NaI water solution (*a*) without laser, (*b*) with 258 nm and 2.7 µJ cm^−2^ laser irradiation and good spatial overlap between the laser and X-ray beam and (*c*) with laser irradiation but imperfect spatial overlap between laser and X-ray beam. (*d*) Specular reflected beam from a 3 *M* NaI solution with a laser fluence of 107 µJ cm^−2^.

The influence of the UV laser on a 0.5 *M* NaI salt-solution–vapour interface was investigated by XRR. The solution was stored in the dark until laser excitation. To electronically excite the sample, it was illuminated with a 258 nm laser beam with 251 fs (FWHM) pulse length and a Gaussian beam shape with a diameter of 15.3 mm (1/e^2^), delivering a fluence of 5.4 µJ cm^−2^. The X-ray beam size was set at 100 µm vertical times 200 µm horizontal at the sample position. The angles of incidence and reflection were set to 0.94° so the specular reflected-ray beam at a *q*_*z*_ position of 0.3 Å^−1^ was monitored. The size of the X-ray footprint on the sample surface was ∼4.7 mm (1/e^2^) along the X-ray direction which was one-third of the laser spot size and the two spots had a good spatial overlap. The shape of the specular reflected X-ray beam observed on the Lambda detector along the *q*_*z*_ (vertical component of the wave transfer vector) and *q*_||_ (horizontal component) direction was investigated before the laser excitation [Fig. 6 ▸(*a*)]. Here, a well defined Gaussian shaped profile is observed along the *q*_*z*_ direction as expected. During laser excitation we observe a strong broadening of the reflected X-ray beam along the *q*_*z*_ direction [Fig. 6 ▸(*b*)] when a good overlap between X-ray and laser beams is achieved. Reducing or increasing the overlap of the X-ray and laser beams causes beam movement of the reflected X-ray beam along the detector. Fig. 6 ▸(*c*) shows the effect of off-setting the laser beam from the centre to the FWHM of the intensity along the sample surface parallel to the incoming X-ray beam to reduce the spatial overlap. Under these conditions, the X-ray beam is hitting the edge of the laser excited surface area. As a result, the reflected X-ray beam moved 13 pixels or 0.011 Å^−1^ (around 4%) along the *q*_*z*_ direction and pronounced asymmetry of the Gaussian shaped X-ray beam is observed because of the misalignment between the X-ray and laser beam. Choosing a higher laser power causes strong peak splitting as shown in Fig. 6 ▸(*d*) for a 3 *M* NaI solution illuminated with a laser fluence of 107 µJ cm^−2^.

### 5.2. Laser induced deformation of the liquid surface

**Figure 7:**
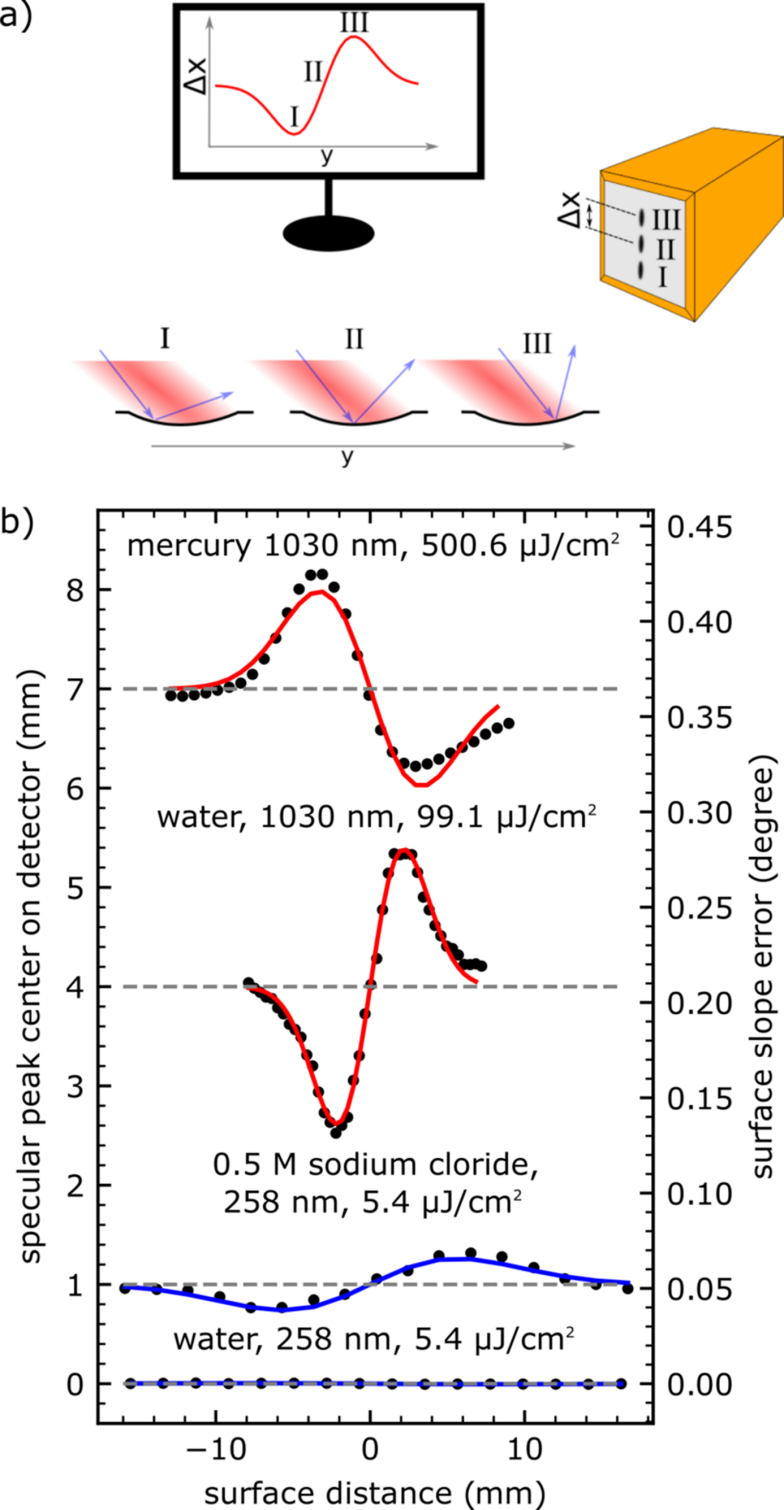
Movement of the specular reflected X-ray beam while scanning along the surface deflection induced by the laser beam profile for mercury, water and 0.5 *M* NaI solution with different laser properties. The measurements were taken at the *q*_*z*_ position 0.3 Å^−1^. Data are shown as symbols and lines indicate best fits of the sample surface profile (Table 1 ▸). The dashed line indicates the position for the X-ray specular peak on the detector without laser irradiation. The individual curves are offset with respect to each other for clarity.

To better understand this sample behaviour when exposed to the laser beam, scans of the surface profile were carried out. Both the sample and X-ray beam were held fixed and the laser spot was scanned along and perpendicular to the X-ray beam direction with a pair of motorized mirrors, as schematically illustrated in Fig. 7 ▸(*a*). These measurements were performed for the NaI and other samples with varying laser configuration. The results are shown in Fig. 7 ▸(*b*), where the vertical X-ray peak position on the detector Δ*x* is plotted against the laser beam position Δ*y* (with Δ*y* = 0 corresponding to the optimal overlap between laser and X-ray beam) and the right-hand axis shows the surface slope error. An anti-symmetrical shift of Δ*x* is observed, most likely coming from a change in the local height and tilt of the sample. This can originate from a dip, bump or standing wave on the liquid surface, as illustrated in Fig. 7 ▸(*a*). Such a laser-induced deformation of the liquid surface may be induced by several effects, including thermal expansion, evaporation, ablation, photon pressure, local changes in surface tension or, most likely, a mixture of several of these. To enable reproducible measurements at the liquid interface, these deflections were investigated in more detail and stable settings were identified. A change in the surface profile results in variations of the reflection angle as a function of the lateral position of the beam on the sample and, consequently, causes X-ray beam movement on the detector. From a careful evaluation of the deflection, the surface profile can be determined. Using the local gradient to calculate the incident angle α for a Gaussian shaped protrusion on a flat surface, the relative movement of the reflected X-ray beam on the detector Δ*x* can be calculated by
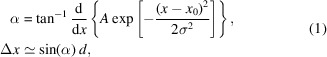
where *d* is the distance between the detector and the sample position. By fitting the Gaussian parameters amplitude *A*, position *x*_0_ and sigma σ to our measured data the shape of the surface disturbance can be estimated.

The calculated fits (solid line) along with the measured data (black dots) are shown in Fig. 7 ▸(*b*) and all parameter are listed in Table 1 ▸. The phenomena could be observed not only for the NaI solution interface excited with a 258 nm laser beam but also from many other liquid samples tested. However, as shown in Fig. 7 ▸, no effect was observed on the pure water surface with the same laser parameter as employed for the NaI sample (258 nm, 5.4 µJ cm^−2^), where a surface slope error of 0.016 ± 0.002° was observed.

Deformation of the sample surface was also found upon purely thermal excitation by infrared laser light. This is illustrated in Fig. 7 ▸ for the case of liquid mercury under H_2_ gas and for pure water under nitro­gen atmosphere. Here the Hg and water sample were excited with a 1030 nm laser beam with a fluence of 450.6 and 89.2 µJ cm^−2^, respectively, and a laser spot size of 6.6 mm (1/e^2^). The X-ray beam parameters were the same as described in Section 5.1. In both cases we could observe a Gaussian shaped surface displacement with a surface slope error of 0.060 ± 0.001° and 0.070 ± 0.001° for Hg and water, respectively. Surprisingly, the direction of the surface deflection on mercury was opposite to that on water. This indicates a thermal expansion leading to a bump on the mercury surface, while on water a dip is formed indicating that other additional effects are decisive. To further determine the effect on the water and mercury surface, some calculations regarding absorption photon pressure and thermal expansion were carried out, as detailed in the supporting information. The observed dip could be due to the evaporation of a small liquid volume in the area of the laser spot. However, even by assuming full absorption of the laser energy by the liquid, the time needed to evaporate the water from the bump would be more than one order of magnitude larger than the observed time for the formation of the dip, which is less than 1 s (see below). Also, the estimated depression of the surface by the photon pressure is orders of magnitudes below the observed effect. A further explanation could be a local change of the surface tension, which would lead to a similar phenomenon to the Marangoni effect. Since the surface tension would only change in the region of the laser illumination, an equilibrium with a complicated fluid dynamic would form. Further investigations are in progress, including alternative approaches as described by Jeng *et al.* (1998 ▸).

**Figure 8:**
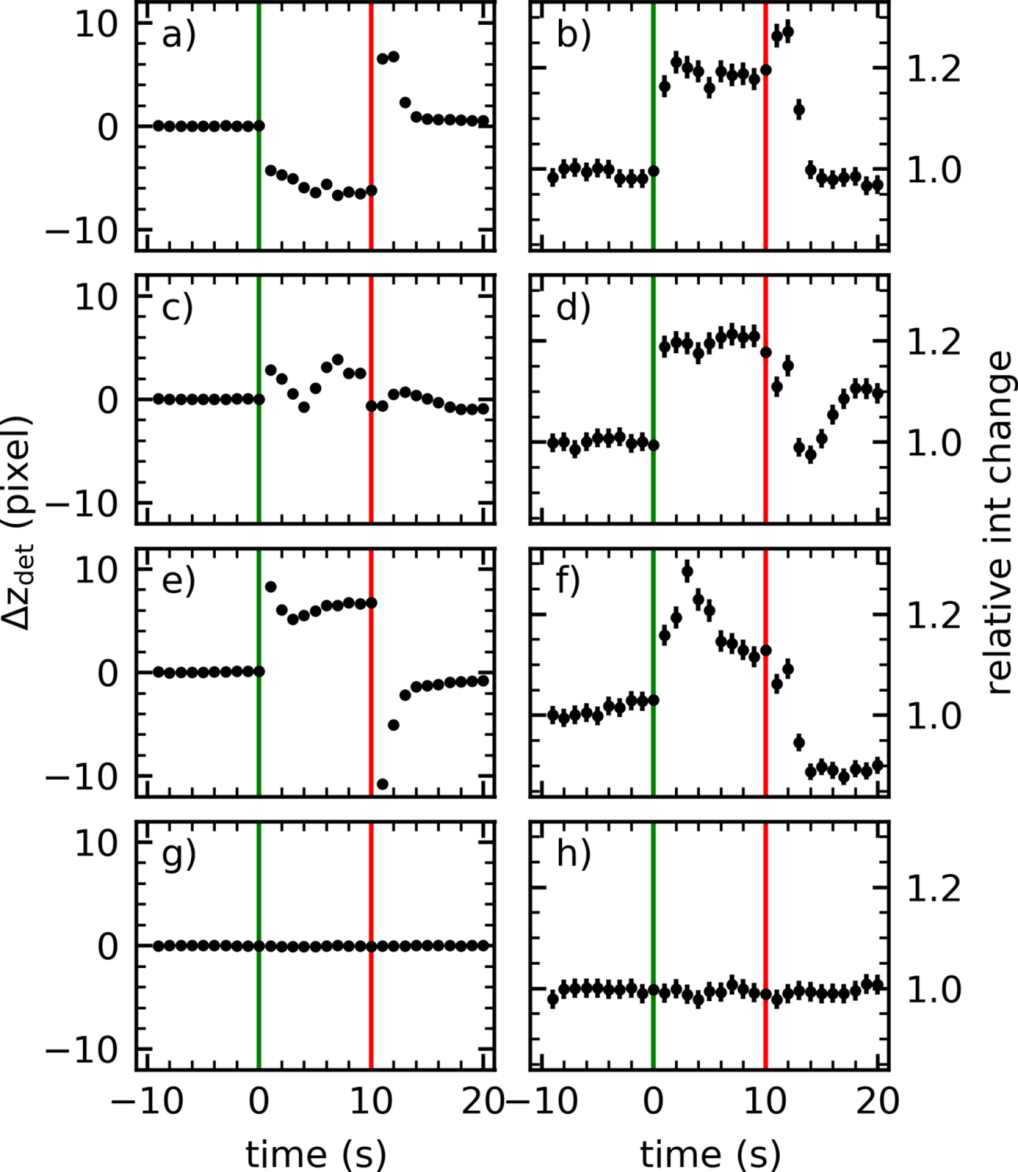
NaI: the specular reflected beam position on the detector (left) and specular reflected intensity (right) at *q*_*z*_ = 0.3 Å^−1^ for a 0.5 *M* NaI solution at different laser/X-ray overlap positions, maxima (*a*, *b*), middle turning points (*c*, *d*) and minima (*e*, *f*), along the trajectory shown in Fig. 7 ▸(*b*). (*g*, *h*) Corresponding data for water. A 258 nm laser beam with a diameter of 15.3 mm and a fluence of 5.4 µJ cm^−2^ was used. The laser was switched on at *t* = 0 s (green line) and switched off at *t* = 10 s (red line). Measurements are averaged over 1 s.

To further clarify the influence of the laser on the liquid surface profile, we performed for 0.5 *M* NaI solution more detailed studies of the evolution of the signal on the time scale of seconds after switching the laser irradiation on and off again. Such studies are an important prerequisite for XRR under optical excitation and pump–probe measurements, because curved surfaces may influence the measured signal and distort the results. Measurements on curved surfaces are possible but require careful data treatment (Festersen *et al.*, 2018 ▸; Konovalov *et al.*, 2022 ▸). In the present case, we have the added complication of confirming the correct spatial overlap between laser and X-rays for the case of a liquid sample. Fig. 8 ▸ shows the intensity changes of a 0.5 *M* NaI solution upon laser excitation with a 258 nm laser beam with a diameter of 15.3 mm and a fluence of 5.4 µJ cm^−2^ at three different overlap positions between laser and X-rays. The measurements were taken at the maxima [Figs. 8(*a*) and 8(*b*) ▸], middle inflection point [Figs. 8(*c*) and 8(*d*) ▸] and minima [Figs. 8(*e*) and 8(*f*) ▸] along the trajectory shown in Fig. 7 ▸. Similar effects are seen for 3 *M* NaI solution illuminated with a laser fluence of 107 µJ cm^−2^, as shown in Fig. S7.

Despite various deviations of the reflected beam position, an increase in the intensity of roughly 20% can be observed in all three cases upon starting laser excitation (vertical green line). These observations indicate that the measured X-ray signal is independent of the surface profile. To investigate whether the measured increase in intensity is attributable to excitation of the NaI solution, the same experiment was carried out on pure water [Figs. 8 ▸(*g*) and 8(*h*)]. In agreement with Fig. 7 ▸, neither a change in the specular reflected beam nor a change in the intensity was observed for water at *q*_*z*_ = 0.3 Å^−1^. Sauer *et al.* (2004 ▸) and Marin *et al.* (2019 ▸) showed absorption for iodine and no absorption for hydroxide at 248 nm. Based on their results, we expect a higher absorption from iodine as compared with hydroxide in the water sample at 258 nm. This higher absorption could explain the intensity increase. If the absorbed energy is converted to heat, it would increase the capillary wave roughness giving a reduction in the specular reflected intensity. Since an intensity increase is observed, other effects are dominant here. Ion segregation at the NaI solution’s surface as predicted by Jungwirth & Tobias (2006 ▸) could explain the increase in intensity. Furthermore, it has been shown that via photoexcitation of inorganic anions, such as iodide, free hydrated electrons are generated (Sauer *et al.*, 2004 ▸; Iwata *et al.*, 1993 ▸; Lian *et al.*, 2006 ▸). These hydrated electrons most likely change the surface structure and influence the iodine at the interface (*i.e.* enrichment). Further investigations and simulations are required to clarify this. During these experiments an ageing of the iodine sample was observed. These ageing effects have already been described by Rahn (1993 ▸), Yeo & Choi (2009 ▸) and Marin *et al.* (2019 ▸). The ageing is caused, among other processes, by the formation of triiodide, noticeable as yellowish discolouration on the sample surface. This occurrence influenced and diminished the observed effects by altering the chemical composition at the liquid surface. Nevertheless, reproducible measurements were possible within 20 minutes to hours depending on the amount of laser excitation and sample concentration. The sample was exchanged upon discolouration.

### 5.3. Surface structure of photoexcited water and NaI solution

**Figure 9:**
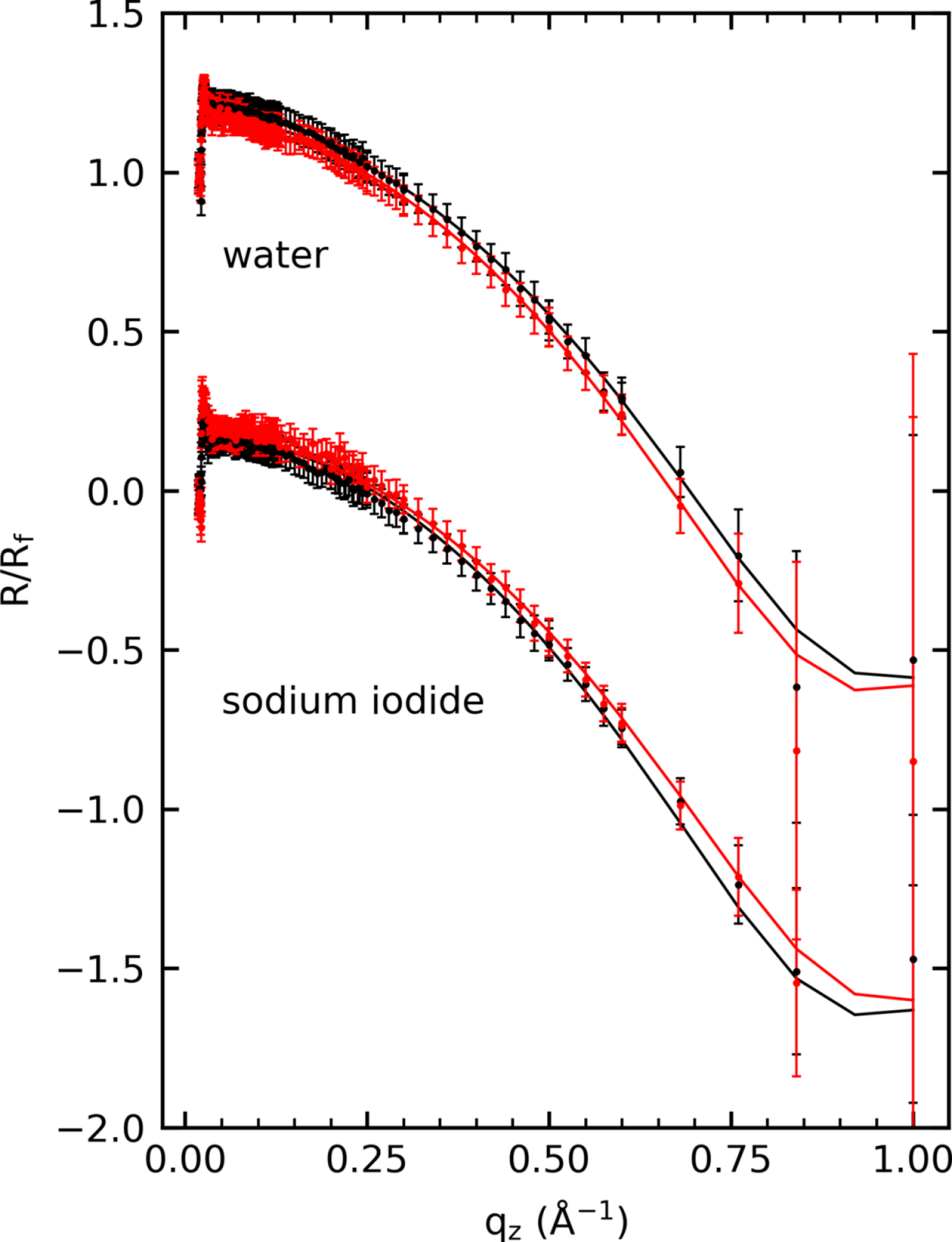
Static XRR measurements with the reflected intensity normalized by the Fresnel intensity (*R*/*R*_f_) and the associated fits are shown for water and 0.5 *M* NaI solution with (red) and without (black) laser excitation. A 258 nm laser beam with a diameter of 15.3 mm and a fluence of 5.4 µJ cm^−2^ was used to continuously illuminate the sample.

To determine the influence of the photoexcitation on the microscopic surface structure, X-ray reflectivity measurements were performed. Fig. 9 ▸ shows the obtained XRR for the 0.5 *M* NaI solution and water with and without laser excitation at 258 nm. As indicated by the time scans in Fig. 8 ▸, an increase in the reflected intensity for the NaI solution upon irradiation is observed. Fitting the XRR curve with a simple roughness model without layering as described by Braslau *et al.* (1985 ▸) and Pershan & Schlossman (2012 ▸) indicates that the roughness decreased from 2.49 ± 0.02 Å to 2.40 ± 0.02 Å during laser irradiation. In contrast, the water XRR exhibits an increase in roughness from 2.41 ± 0.02 Å to 2.74 ± 0.02 Å. Even if the changes are small, these results indicate an opposite effect for the two samples. The increase in the water roughness can be explained by an increase in the (local) temperature due to absorption or a small distortion of the water surface due to the laser. This increase in roughness could not be found in the previously shown intensity transients [Fig. 8 ▸(*h*)], which may be explained by the low influence of roughness on the XRR at the low *q*_*z*_ values of 0.3 Å^−1^. The reduced roughness seen for the NaI solution indicates interesting changes in the surface structure. Temperature-induced increases in capillary roughness do not seem to play an overriding role or may be overcompensated by other effects. The reduced roughness could hint at an increase in the surface tension, which could be introduced by an increased salt concentration at the surface due to laser excitation. This would be in accordance with theoretical studies (Jungwirth & Tobias, 2006 ▸).

Investigation of electronic and thermal excitations effects on nanosecond to sub-nanosecond time scale pump–probe measurements on NaI solutions were observed but not in a reproducible manner. Sub-nanosecond effects could only be observed in some first scans on fresh NaI solutions, most likely due to sample ageing. Such measurements require very long counting times since the signal is very low in timing mode as only one X-ray bunch from 40 is used as described above in Section 3.1. Thus, though counter-intuitive, the total exposure of the solution to the laser is much longer for measurements on faster time scales resulting in sample ageing. The laser exposure for a typical pump–probe measurement can take up to 10 min in comparison with ‘burst mode’ measurements where it can be reduced to 10 s. To solve this issue of reproducibility, a flow cell is in development. In the case of liquid mercury, highly reproducible pump–probe data could be collected on the sub-nanosecond time scale (a dedicated publication is in progress to describe this complex system).

## 6. Conclusion

The LISA diffractometer at PETRA III has been upgraded with a laser-pump–X-ray-probe providing the possibility of a time resolution in the range of the X-ray length of σ_total_ = 38 ± 6 ps. Though further tests are required on liquid interfaces, this time scale was confirmed by measuring the thermal expansion of the solid Bi (111) Bragg peak following the excitation by a laser pulse.

The LISA setup is tailored to investigate liquid interfaces. Significant experimental issues could be overcome to describe the laser induced deformation of the liquid surface. Laser induced changes of the shape of liquid interfaces are investigated and modelled, as a prerequisite for optimized experimental studies on liquids. It is also shown that laser light can have a measurable influence on liquid surfaces and that reproducible experiments are possible currently on longer time scales. Preliminary X-ray reflectivity measurements on an NaI solution interface illustrate that UV-laser excitation can induce non-thermal effects that can be investigated with the new setup. Another issue is laser beam damage of liquid solutions which combined with weak scattering hampered reproducibility on the shortest time scales. Further tests with a flow cell are required to counter this problem.

The laser setup is also available for studies of other solid and liquid systems at the Kohzu diffractometer at the P08 beamline. Despite encountering experimental challenges and solution degradation, this set-up sets the foundation for further exploration of effects across all temporal domains at liquid surfaces including liquid metals. The presented work will pave the way for time-resolved investigations of surface structural dynamics at liquid interfaces down to picosecond time scales.

## 7. Related literature

The following references, not cited in the main body of the paper, have been cited in the supporting information: Johnson & Christy (1972 ▸); Weber (2018 ▸).

## Supplementary Material

Supporting information file. DOI: 10.1107/S1600577524003400/ju5055sup1.pdf

## Figures and Tables

**Table 1:** Laser parameter and fit values of the Gaussian shaped laser induced surface deflection

Sample	Laser wavelength (nm)	Laser diameter (1/e^2^ )(mm)	Laser fluence (µJ cm^−2^)	Diameter (1/e^2^) (mm)	Amplitude (µm)
Mercury	1030	6.6	450.6	14.8	5.5
Water	1030	6.6	89.2	8.7	−4.5
NaI 0.5 *M*	258	15.3	5.4	22.4	−2.2
Water	258	15.3	5.4		0

## References

[bb1] AlHassan, A., Lähnemann, J., Leake, S., Küpers, H., Niehle, M., Bahrami, D., Bertram, F., Lewis, R. B., Davtyan, A., Schülli, T. U., Geelhaar, L. & Pietsch, U. (2020). *Nanotechnology*, **31**, 214002. 10.1088/1361-6528/ab7590 32050166

[bb2] Antonowicz, J., Zalden, P., Sokolowski-Tinten, K., Georgarakis, K., Minikayev, R., Pietnoczka, A., Bertram, F., Chaika, M., Chojnacki, M., Dłużewski, P., Fronc, K., Greer, A. L., Jastrzębski, C., Klinger, D., Lemke, C., Magnussen, O. M., Murphy, B., Perumal, K., Ruett, U., Warias, J. & Sobierajski, R. (2021). *J. Alloys Compd.* **887**, 161437.

[bb3] Balewski, K., Bieler, M., Wanzenberg, R., Keil, J., Kling, A. & Sahoo, G. K. (2011). *Proceedings of the 2nd International Particle Accelerator Conference (IPAC2011)*, 4–9 September 2011, San Sebastián, Spain, pp. 2948–2950. THPC020.

[bb4] Benjamin, I. (2006). *Chem. Rev.* **106**, 1212–1233. 10.1021/cr040362f 16608178

[bb5] Biasin, E., Fox, Z. W., Andersen, A., Ledbetter, K., Kjaer, K. S., Alonso-Mori, R., Carlstad, J. M., Chollet, M., Gaynor, J. D., Glownia, J. M., Hong, K., Kroll, T., Lee, J. H., Liekhus-Schmaltz, C., Reinhard, M., Sokaras, D., Zhang, Y., Doumy, G., March, A. M., Southworth, S. H., Mukamel, S., Gaffney, K. J., Schoenlein, R. W., Govind, N., Cordones, A. A. & Khalil, M. (2021). *Nat. Chem.* **13**, 343–349. 10.1038/s41557-020-00629-3 33589787

[bb6] Biasin, E., van Driel, T. B., Kjaer, K. S., Dohn, A. O., Christensen, M., Harlang, T., Chabera, P., Liu, Y., Uhlig, J., Pápai, M., Németh, Z., Hartsock, R., Liang, W., Zhang, J., Alonso-Mori, R., Chollet, M., Glownia, J. M., Nelson, S., Sokaras, D., Assefa, T. A., Britz, A., Galler, A., Gawelda, W., Bressler, C., Gaffney, K. J., Lemke, H. T., Møller, K. B., Nielsen, M. M., Sundström, V., Vankó, G., Wärnmark, K., Canton, S. E. & Haldrup, K. (2016). *Phys. Rev. Lett.* **117**, 013002. 10.1103/PhysRevLett.117.013002 27419566

[bb7] Braslau, A., Deutsch, M., Pershan, P. S., Weiss, A. H., Als-Nielsen, J. & Bohr, J. (1985). *Phys. Rev. Lett.* **54**, 114–117. 10.1103/PhysRevLett.54.114 10031258

[bb8] Bugayev, A. & Elsayed-Ali, H. E. (2019). *J. Phys. Chem. Solids*, **129**, 312–316.

[bb9] Burian, M., Marmiroli, B., Radeticchio, A., Morello, C., Naumenko, D., Biasiol, G. & Amenitsch, H. (2020). *J. Synchrotron Rad.* **27**, 51–59. 10.1107/S1600577519015728 PMC6927520 31868736

[bb10] Chandran, S., Dold, S., Buvignier, A., Krannig, K.-S., Schlaad, H., Reiter, G. & Reiter, R. (2015). *Langmuir*, **31**, 6426–6435. 10.1021/acs.langmuir.5b01212 26000718

[bb11] Chin, J. W., Aouadi, K., Haight, M. R., Hughes, W. L. & Nguyen, T. (2001). *Polym. Compos.* **22**, 282–297.

[bb12] Collet, E., Lorenc, M., Cammarata, M., Guérin, L., Servol, M., Tissot, A., Boillot, M.-L., Cailleau, H. & Buron–Le Cointe, M. (2012). *Chem. A Eur. J.* **18**, 2051–2055. 10.1002/chem.201103048 22246788

[bb13] Dallari, F., Jain, A., Sikorski, M., Möller, J., Bean, R., Boesenberg, U., Frenzel, L., Goy, C., Hallmann, J., Kim, Y., Lokteva, I., Markmann, V., Mills, G., Rodriguez-Fernandez, A., Roseker, W., Scholz, M., Shayduk, R., Vagovic, P., Walther, M., Westermeier, F., Madsen, A., Mancuso, A. P., Grübel, G. & Lehmkühler, F. (2021). *IUCrJ*, **8**, 775–783. 10.1107/S2052252521006333 PMC8420773 34584738

[bb14] Eisenthal, K. B. (1996). *Chem. Rev.* **96**, 1343–1360. 10.1021/cr9502211 11848793

[bb15] Elsen, A., Festersen, S., Runge, B., Koops, C. T., Ocko, B. M., Deutsch, M., Seeck, O. H., Murphy, B. M. & Magnussen, O. M. (2013). *Proc. Natl Acad. Sci. USA*, **110**, 6663–6668. 10.1073/pnas.1301800110 PMC3637733 23553838

[bb16] Elsen, A., Murphy, B. M., Ocko, B. M., Tamam, L., Deutsch, M., Kuzmenko, I. & Magnussen, O. M. (2010). *Phys. Rev. Lett.* **104**, 105501. 10.1103/PhysRevLett.104.105501 20366434

[bb17] Enquist, H., Jurgilaitis, A., Jarnac, A., Bengtsson, Å. U. J., Burza, M., Curbis, F., Disch, C., Ekström, J. C., Harb, M., Isaksson, L., Kotur, M., Kroon, D., Lindau, F., Mansten, E., Nygaard, J., Persson, A. I. H., Pham, V. T., Rissi, M., Thorin, S., Tu, C.-M., Wallén, E., Wang, X., Werin, S. & Larsson, J. (2018). *J. Synchrotron Rad.* **25**, 570–579. 10.1107/S1600577517017660 PMC5829682 29488939

[bb18] Esmail, A. R., Bugayev, A. & Elsayed-Ali, H. E. (2013). *J. Phys. Chem. C*, **117**, 9035–9041.

[bb19] Fadaly, E. M. T., Dijkstra, A., Suckert, J. R., Ziss, D., van Tilburg, M. A. J., Mao, C., Ren, Y., van Lange, V. T., Korzun, K., Kölling, S., Verheijen, M. A., Busse, D., Rödl, C., Furthmüller, J., Bechstedt, F., Stangl, J., Finley, J. J., Botti, S., Haverkort, J. E. M. & Bakkers, E. P. A. M. (2020). *Nature*, **580**, 205–209. 10.1038/s41586-020-2150-y 32269353

[bb20] Festersen, S., Hrkac, S. B., Koops, C. T., Runge, B., Dane, T., Murphy, B. M. & Magnussen, O. M. (2018). *J. Synchrotron Rad.* **25**, 432–438. 10.1107/S1600577517018057 29488923

[bb21] Fradin, C., Braslau, A., Luzet, D., Smilgies, D., Alba, M., Boudet, N., Mecke, K. & Daillant, J. (2000). *Nature*, **403**, 871–874. 10.1038/35002533 10706279

[bb22] Fu, L., Bienenstock, A. & Brennan, S. (2009). *J. Chem. Phys.* **131**, 234702. 10.1063/1.3273874 20025337

[bb23] Fukuyama, Y., Yasuda, N., Kim, J., Murayama, H., Ohshima, T., Tanaka, Y., Kimura, S., Kamioka, H., Moritomo, Y., Toriumi, K., Tanaka, H., Kato, K., Ishikawa, T. & Takata, M. (2008). *Rev. Sci. Instrum.* **79**, 045107. 10.1063/1.2906232 18447552

[bb24] Haddad, J., Pontoni, D., Murphy, B. M., Festersen, S., Runge, B., Magnussen, O. M., Steinrück, H.-G., Reichert, H., Ocko, B. M. & Deutsch, M. (2018). *Proc. Natl Acad. Sci. USA*, **115**, E1100–E1107. 10.1073/pnas.1716418115 PMC5819424 29358372

[bb25] Hobley, J., Oori, T., Kajimoto, S., Gorelik, S., Hönig, D., Hatanaka, K. & Fukumura, H. (2008). *Appl. Phys. A*, **93**, 947–954.

[bb26] Hyman, A. A., Weber, C. A. & Jülicher, F. (2014). *Annu. Rev. Cell Dev. Biol.* **30**, 39–58. 10.1146/annurev-cellbio-100913-013325 25288112

[bb27] Issenmann, D., Ibrahimkutty, S., Steininger, R., Göttlicher, J., Baumbach, T., Hiller, N., Müller, A.-S. & Plech, A. (2013). *J. Phys. Conf. Ser.* **425**, 092007.

[bb28] Iwata, A., Nakashima, N., Kusaba, M., Izawa, Y. & Yamanaka, C. (1993). *Chem. Phys. Lett.* **207**, 137–142.

[bb29] Jeng, U.-S., Esibov, L., Crow, L. & Steyerl, A. (1998). *J. Phys. Condens. Matter*, **10**, 4955–4962.

[bb30] Johnson, P. B. & Christy, R. W. (1972). *Phys. Rev. B*, **6**, 4370–4379.

[bb31] Jonge, N. de & Ross, F. M. (2011). *Nat. Nanotechnol.* **6**, 695–704. 10.1038/nnano.2011.161 22020120

[bb32] Jungwirth, P. & Tobias, D. J. (2006). *Chem. Rev.* **106**, 1259–1281. 10.1021/cr0403741 16608180

[bb33] Kessler, J., Elgabarty, H., Spura, T., Karhan, K., Partovi-Azar, P., Hassanali, A. A. & Kühne, T. D. (2015). *J. Phys. Chem. B*, **119**, 10079–10086. 10.1021/acs.jpcb.5b04185 26174102

[bb34] Kim, K. H., Späh, A., Pathak, H., Yang, C., Bonetti, S., Amann-Winkel, K., Mariedahl, D., Schlesinger, D., Sellberg, J. A., Mendez, D., van der Schot, G., Hwang, H. Y., Clark, J., Shigeki, O., Tadashi, T., Harada, Y., Ogasawara, H., Katayama, T., Nilsson, A. & Perakis, F. (2020). *Phys. Rev. Lett.* **125**, 076002. 10.1103/PhysRevLett.125.076002 32857536

[bb35] Klute, J., Balewski, K., Delfs, A., Duhme, H. T., Ebert, M., Neumann, R. & Obier, F. (2011). *Proceedings of the 10th European Workshop on Beam Diagnostics and Instrumentation for Particle Accelerators (DIPAC2011)*, 16–18 May 2011, Hamburg, Germany, pp. 494–496. TUPD81.

[bb36] Konovalov, O. V., Belova, V., La Porta, F., Saedi, M., Groot, I. M. N., Renaud, G., Snigireva, I., Snigirev, A., Voevodina, M., Shen, C., Sartori, A., Murphy, B. M. & Jankowski, M. (2022). *J. Synchrotron Rad.* **29**, 711–720. 10.1107/S1600577522002053 PMC9070704 35511004

[bb37] Kraack, H., Deutsch, M., Ocko, B. & Pershan, P. (2003). *Nucl. Instrum. Methods Phys. Res. B*, **200**, 363–370.

[bb38] Ladd-Parada, M., Amann-Winkel, K., Kim, K. H., Späh, A., Perakis, F., Pathak, H., Yang, C., Mariedahl, D., Eklund, T., Lane, T. J., You, S., Jeong, S., Weston, M., Lee, J. H., Eom, I., Kim, M., Park, J., Chun, S. H. & Nilsson, A. (2022). *J. Phys. Chem. B*, **126**, 2299–2307. 10.1021/acs.jpcb.1c10906 PMC8958512 35275642

[bb39] Laulhé, C., Cammarata, M., Servol, M., Miller, R. J. D., Hada, M. & Ravy, S. (2013). *Eur. Phys. J. Spec. Top.* **222**, 1277–1285.

[bb40] Lian, R., Oulianov, D. A., Crowell, R. A., Shkrob, I. A., Chen, X. & Bradforth, S. E. (2006). *J. Phys. Chem. A*, **110**, 9071–9078. 10.1021/jp0610113 16854017

[bb41] Luo, G., Bu, W., Mihaylov, M., Kuzmenko, I., Schlossman, M. L. & Soderholm, L. (2013). *J. Phys. Chem. C*, **117**, 19082–19090.

[bb42] March, A. M., Stickrath, A., Doumy, G., Kanter, E. P., Krässig, B., Southworth, S. H., Attenkofer, K., Kurtz, C. A., Chen, L. X. & Young, L. (2011). *Rev. Sci. Instrum.* **82**, 073110. 10.1063/1.3615245 21806175

[bb43] Marin, T. W., Janik, I. & Bartels, D. M. (2019). *Phys. Chem. Chem. Phys.* **21**, 24419–24428. 10.1039/c9cp03805a 31663553

[bb44] Meng, Q., Qian, S., Ding, J., Li, Q., Zhao, X., Su, B. & Zhang, C. (2022). *Sci. Rep.* **12**, 8144. 10.1038/s41598-022-11858-6 PMC9114126 35581221

[bb45] Menlo Systems (2009). DDS120 Direct Digital Synthesizer, v1.0. User Manual. MenloSystems GmbH, Munich, Germany.

[bb46] Menlo Systems (2014). *SYNCRO–RRE Locking Electronics*, v2.4. User Manual. MenloSystems GmbH, Munich, Germany.

[bb47] Miller, R., Kretzschmar, G. & Dukhin, S. S. (1995). *Dynamics of Adsorption at Liquid Interfaces.* Holanda: Elsevier.

[bb48] Miranda, P. B. & Shen, Y. R. (1999). *J. Phys. Chem. B*, **103**, 3292–3307.

[bb49] Murphy, B. M., Greve, M., Runge, B., Koops, C. T., Elsen, A., Stettner, J., Seeck, O. H. & Magnussen, O. M. (2014). *J. Synchrotron Rad.* **21**, 45–56. 10.1107/S1600577513026192 24365915

[bb50] Navirian, H., Shayduk, R., Leitenberger, W., Goldshteyn, J., Gaal, P. & Bargheer, M. (2012). *Rev. Sci. Instrum.* **83**, 063303. 10.1063/1.4727872 22755618

[bb51] Nepolian, J. V., Siingh, D., Singh, R. P., Gautam, A. S. & Gautam, S. (2021). *Aerosol Sci. Eng.* **5**, 460–477.

[bb52] Nguyen, T. T. & Tran, M.-B. (2018). *SIAM J. Math. Anal.* **50**, 2020–2047.

[bb53] Ni, Y. & Skinner, J. L. (2015). *J. Chem. Phys.* **143**, 014502. 10.1063/1.4923462 26156483

[bb54] Onufriev, A. V. & Izadi, S. (2018). *WIREs Comput. Mol. Sci.* **8**, e1347.

[bb55] Oum, K. W., Lakin, M. J., DeHaan, D. O., Brauers, T. & Finlayson-Pitts, B. J. (1998). *Science*, **279**, 74–76. 10.1126/science.279.5347.74 9417027

[bb56] Pattadar, D., Cheek, Q., Sartori, A., Zhao, Y., Giri, R. P., Murphy, B., Magnussen, O. & Maldonado, S. (2021). *Cryst. Growth Des.* **21**, 1645–1656.

[bb57] Perakis, F., Amann-Winkel, K., Lehmkühler, F., Sprung, M., Mariedahl, D., Sellberg, J. A., Pathak, H., Späh, A., Cavalca, F., Schlesinger, D., Ricci, A., Jain, A., Massani, B., Aubree, F., Benmore, C. J., Loerting, T., Grübel, G., Pettersson, L. G. M. & Nilsson, A. (2017). *Proc. Natl Acad. Sci. USA*, **114**, 8193–8198. 10.1073/pnas.1705303114 PMC5547632 28652327

[bb58] Pershan, P. S. & Schlossman, M. L. (2012). *Liquid Surfaces and Interfaces: Synchrotron X-ray Methods:* Cambridge University Press.

[bb59] Petersen, P. B. & Saykally, R. J. (2006). *Annu. Rev. Phys. Chem.* **57**, 333–364. 10.1146/annurev.physchem.57.032905.104609 16599814

[bb60] Rahn, R. O. (1993). *Photochem. Photobiol.* **58**, 874–880. 10.1111/j.1751-1097.1993.tb04986.x 8310010

[bb61] Reinhard, M., Skoien, D., Spies, J. A., Garcia-Esparza, A. T., Matson, B. D., Corbett, J., Tian, K., Safranek, J., Granados, E., Strader, M., Gaffney, K. J., Alonso-Mori, R., Kroll, T. & Sokaras, D. (2023). *Struct. Dyn.* **10**, 054304. 10.1063/4.0000207 PMC10613086 37901682

[bb62] Rhew, R. C., Miller, B. R. & Weiss, R. F. (2000). *Nature*, **403**, 292–295. 10.1038/35002043 10659844

[bb63] Runge, B., Festersen, S., Koops, C. T., Elsen, A., Deutsch, M., Ocko, B. M., Seeck, O. H., Murphy, B. M. & Magnussen, O. M. (2016). *Phys. Rev. B*, **93**, 165408.

[bb64] Sartori, A., Giri, R. P., Fujii, H., Hövelmann, S. C., Warias, J. E., Jordt, P., Shen, C., Murphy, B. M. & Magnussen, O. M. (2022). *Nat. Commun.* **13**, 5421. 10.1038/s41467-022-32932-7 PMC9477831 36109498

[bb65] Sauer, M. C., Crowell, R. A. & Shkrob, I. A. (2004). *J. Phys. Chem. A*, **108**, 5490–5502.

[bb66] Schlie, M. (2013). PhD Thesis, University of Hamburg, Germany.

[bb67] Schlossman, M. L. (2002). *Curr. Opin. Colloid Interface Sci.* **7**, 235–243.

[bb68] Seeck, O. H., Deiter, C., Pflaum, K., Bertam, F., Beerlink, A., Franz, H., Horbach, J., Schulte-Schrepping, H., Murphy, B. M., Greve, M. & Magnussen, O. (2012). *J. Synchrotron Rad.* **19**, 30–38. 10.1107/S0909049511047236 22186641

[bb69] Sega, M. & Dellago, C. (2017). *J. Phys. Chem. B*, **121**, 3798–3803. 10.1021/acs.jpcb.6b12437 28218854

[bb70] Seo, J., García-Mayoral, R. & Mani, A. (2018). *J. Fluid Mech.* **835**, 45–85.

[bb71] Shen, C., Kirchhof, R. & Bertram, F. (2022). *J. Phys. Conf. Ser.* **2380**, 012047.

[bb72] Stefaniu, C., Brezesinski, G. & Möhwald, H. (2014). *Adv. Colloid Interface Sci.* **208**, 197–213. 10.1016/j.cis.2014.02.013 24612663

[bb73] Tamarat, P., Maali, A., Lounis, B. & Orrit, M. (2000). *J. Phys. Chem. A*, **104**, 1–16. 10.1021/jp992505l 36411678

[bb74] Wang, H., Yu, C., Wei, X., Gao, Z., Xu, G.-L., Sun, D.-R., Li, Z., Zhou, Y., Li, Q.-J., Zhang, B.-B., Xu, J.-Q., Wang, L., Zhang, Y., Tan, Y.-L. & Tao, Y. (2017). *J. Synchrotron Rad.* **24**, 667–673. 10.1107/S1600577517003277 28452759

[bb75] Weber, M. J. (2018). *Handbook of Optical Materials.* CRC Press.

[bb76] Wulff, M., Plech, A., Eybert, L., Randler, R., Schotte, F. & Anfinrud, P. (2003). *Faraday Disc.* **122**, 13–26. 10.1039/b202740m 12555847

[bb77] Yeo, J. & Choi, W. (2009). *Environ. Sci. Technol.* **43**, 3784–3788. 10.1021/es900602n 19544888

[bb78] Yong, H., Xu, X., Ruddock, J. M., Stankus, B., Carrascosa, A. M., Zotev, N., Bellshaw, D., Du, W., Goff, N., Chang, Y., Boutet, S., Carbajo, S., Koglin, J. E., Liang, M., Robinson, J. S., Kirrander, A., Minitti, M. P. & Weber, P. M. (2021). *Proc. Natl Acad. Sci. USA*, **118**, e2021714118. 10.1073/pnas.2021714118 PMC8126834 33947814

[bb79] Zhang, H., Eickemeyer, F. T., Zhou, Z., Mladenović, M., Jahanbakhshi, F., Merten, L., Hinderhofer, A., Hope, M. A., Ouellette, O., Mishra, A., Ahlawat, P., Ren, D., Su, T.-S., Krishna, A., Wang, Z., Dong, Z., Guo, J., Zakeeruddin, S. M., Schreiber, F., Hagfeldt, A., Emsley, L., Rothlisberger, U., Milić, J. V. & Grätzel, M. (2021*b*). *Nat. Commun.* **12**, 3383. 10.1038/s41467-021-23566-2 PMC8185086 34099667

[bb80] Zhang, Y., Sprittles, J. E. & Lockerby, D. A. (2021*a*). *J. Fluid Mech.* **915**, A135.

