# Supplementary material for: The laser pump X-ray probe system at LISA P08 PETRA III
Source: J Synchrotron Radiat. 2024 Jun 6;31(Pt 4):779–90. doi: 10.1107/S1600577524003400 (PMC11226150; doi:10.1107/S1600577524003400)
Supplement: Supplementary file 1 [file s-31-00779-sup1.pdf]

JOURNAL OF
SYNCHROTRON
RADIATION

Volume 31 (2024)

Supporting information for article:

The laser pump X-ray probe system at LISA P08 PETRA III

Jonas Erik Warias, Lukas Petersdorf, Svenja Carolin Hövelmann, Rajendra Prasad Giri, Christoph Lemke, Sven Festersen, Matthias Greve, Philippe Mandin, Damien LeBideau, Florian Bertram, Olaf Magnus Magnussen and Bridget Mary Murphy

S1. Parameter laser system

Table S1 Parameter laser system.

Laser module	Wavelength (nm)	Repetition rate (MHz)	Max. power (W)	Max. pulse energy (µJ)	Pulse length (ps)
Pharos Laser	1030	0.1-1.0	15	150	0.23-12
Hiro 2nd, 3rd, 4th harmonic generator	515<br>343<br>258	~0.1	7.5<br>4.5<br>1.2	75<br>45<br>12	0.23
Orpheus optical parametric amplifier	210 - 2600	1.04	Fig. S1	Fig. S1	0.23

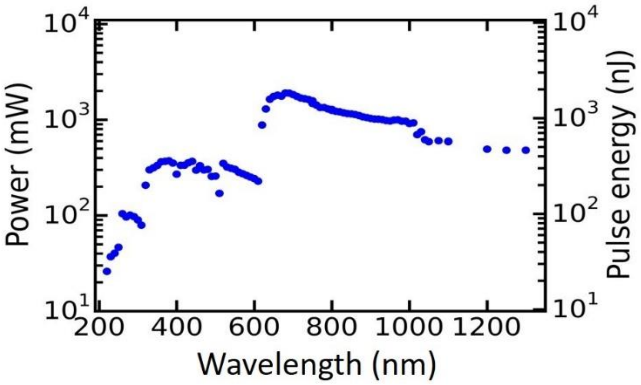

Figure S1 Wavelength dependence of the output power and pulse energy of the Orpheus optical parametric amplifier.

S2. Image of the mercury laser cell

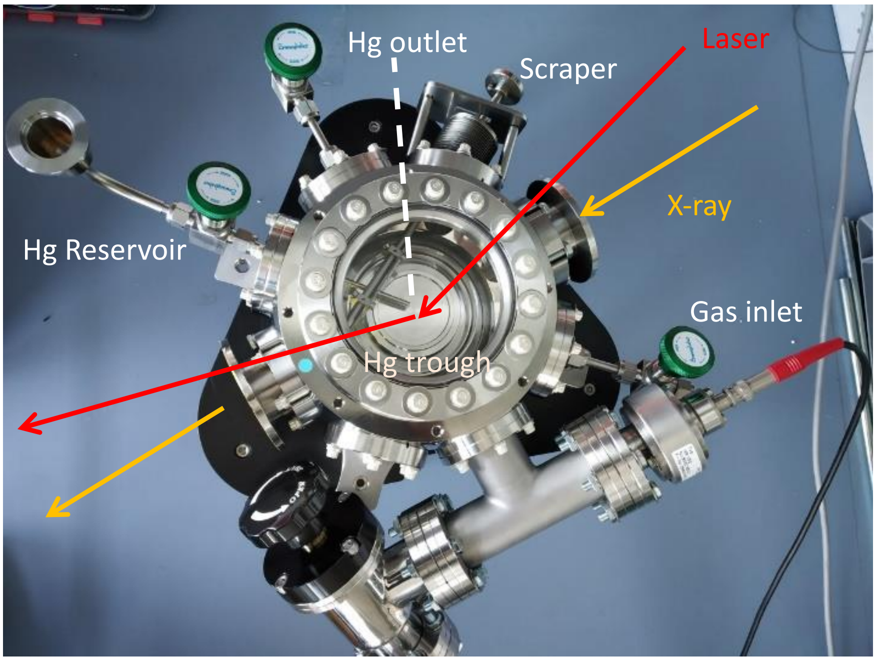

Figure S2 Image of the Hg cell and schematic drawing of the X-ray and laser path and naming of cell parts as outlined in the main paper.

S3. Image of the water and aqueous salt laser cell

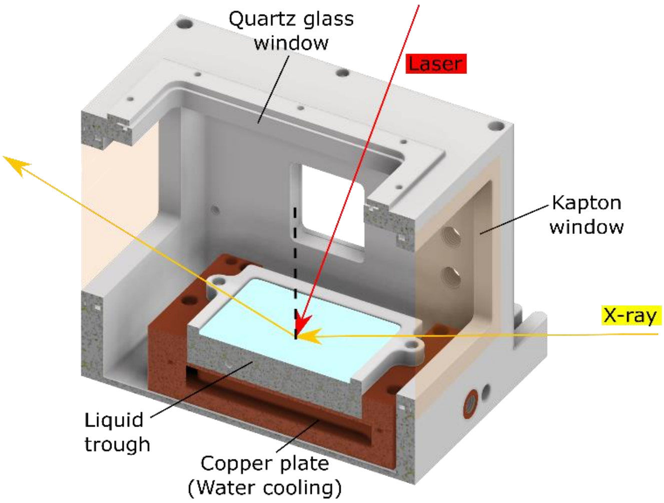

Figure S3 Cut image of the laser cell for measurements on water and NaI solutions as outlined in the main paper. The cell was and is undergoing continuously improvements.

S4. Estimations of the observed surface disturbance

To better understand the laser beam-induced mechanical disturbance of the surface, some simple estimations for pure water and mercury were made. The observed dip on the water surface could be related to the thermal evaporation of water. First, the volume of the observed Gaussian shaped distorted surface was calculated to be

$$Vol = 2\pi A\sigma_x\sigma_y. \quad (1)$$

where A is the absolute amplitude and σ is the standard deviation with σ_x along the X-ray beam (x direction) and σ_y perpendicular to the X-ray beam (y direction). σ_x was measured as described in the manuscript and is listed along with other relevant values in Table S2. $\sigma_y = 0.84\sigma_x$ was used as a lower estimation of the volume due to the asymmetric shape of the incoming laser beam at an angle of 30 degree. To evaporate the observed volume of the dip on the water surface the specific heat capacity (C) and the enthalpy of vaporization (H_{vap}) need to be exceeded. The heat capacity is given by:

$$C = \frac{\Delta Q}{m\Delta T}. \quad (2)$$

ΔQ represents the amount of heat needed to raise the sample temperature by ΔT and m the mass of the sample. The required energy (E_{vap}) to heat the sample and evaporate the liquid volume can be calculated by assuming an absorbed percentage (ϑ) of the average incoming laser energy E_L as follows

$$E_{vap} = \frac{m(\Delta TC + H_{vap})}{\vartheta E_L}. \quad (3)$$

To evaporate the observed volume of 1.1e-10 m^3 an energy of 0.23 J is necessary. The laser provides 2 J/s with the used beam specification. Since the heat absorption at 1030 nm is below 1% within the 4.5 µm amplitude of the observed dip, (Weber, 2018) the available energy becomes 0.24e-3 J which is much smaller than the calculated energy required for the evaporation. This suggests that the energy dumped in the surface region is insufficient for the evaporation of the observed volume. Additional computer simulations similar to the presented mercury simulations (Fig. S5) show a maximum

heating of the laser irradiated bulk water by less than 20 degree. Since we are using a pulsed laser system with relatively high pulse energy, evaporation similar to ablation effects seen for metals could happen. Nevertheless, water is evaporating but it solely does not explain the observed dip on the water surface.

Another possibility could be the repression of the water surface by the photon pressure. The photon pressure p_{St} for monochromatic light is given by

$$p_{St} = \frac{h\upsilon \cos\theta}{c} \frac{dN}{dtdA}, \tag{4}$$

with the Planck constant h, the light frequency υ, the speed of light c, the incident angle θ, and the number of photons per unit time and area $dN/dtdA$. For an upper estimation we can ignore the angle of incident and simply calculate the effective force by using the radiant flux Φ from our laser, equivalent to the listed laser energy E_L

$$F_R = \frac{h\upsilon}{c} \frac{dN}{dt} = \frac{\Phi}{c}. \tag{5}$$

The main repulsive forces from the liquid surface are the surface tension and buoyancy F_B. The buoyancy is given by

$$F_B = V\rho g, \tag{6}$$

using the Volume V, density ρ and gravitational constant g. The photon pressure is in the range of 7e-9 N when assuming full absorption. The opposing buoyancy is in the range of 1e-6 N similar to the surface tension in the range of 1e-7 N. In both cases the photon pressure is much smaller and would not contribute to the observed dip.

The observed bump for mercury is most likely related to the local thermal expansion of the mercury. The linear and volumetric thermal expansion coefficients are defined respectively as

$$\alpha_L = \frac{1}{L}\frac{dL}{dT} \text{ and } \alpha_V = \frac{1}{V}\frac{dV}{dT} \quad (7)$$

where α_L and α_V are the linear and volumetric thermal expansion coefficients respectively. L, V and T are respectively the length, volume and temperature and dL, dV and dT are their respective changes. Since the diameter of the mercury containing inner sample cell was 50 mm which is ~ 10-fold higher than the depth (5 mm) and ~ 8-fold higher than the laser illuminated portion (6.6 mm, $1/e^2$) we have calculated the thermal expansion only along the depth from a simplified approximation. The reported linear thermal expansion coefficient of mercury is 60.4 µm/(m K). By approximating a uniform temperature difference (dT) of 40 K (Fig. S5) along the depth (5 mm) the value of thermal expansion along an axis normal to the sample surface has been calculated to be ~ 12.1 µm.

Table S2 Estimated values in relation to the observed surface disturbances.

sample	A (µm)	σ_x (m)	Vol (m³)	E_L (W)	E_{abs} (W)	H_{vap} (J/kg)	C (J/kg K)	E_{vap} (J)	F_R (N)	F_B (N)	A_{calc} (µm)
H_2O	-4.5	2.17e-3	-1.1e-10	2	-0.24e-3	2.3e6	4.2e3	0.23	7.0e-9	1e-6	
Hg	5.5	3.70e-3	4.0e-10	10	10	0.3e6	1.4e2		3.3e-8		12.1

All element properties were taken from www.nist.gov.

S5. Steady state temperature modelling

The goal of this modelling is to describe the spatial and temporal evolution of temperature of a thin (~ 5 mm) layer of liquid mercury.

S5.1. Heat transfer schemes

Here, three ways of heat transfer have been considered as described in Fig. S4. The sample is in contact with the air, i.e., convection takes place and the sample loses heat. The Teflon substrate is an opaque solid so heat is transported by conduction through this material. The liquid Hg sample in this experiment is thin (~ 5mm) and the liquid do not move so we can assume that the heat is transported mainly by conduction. The laser beam is considered as a radiative heat source. The laser beam hits the sample with angle of incidence of 20° compared to the normal of the surface. Most of the energy is

dumped at the surface of mercury due to its high thermal absorption coefficient. Even though a simulation has been done with a small Teflon cell with significantly lower heat conductivity compared to the finally used stainless steel cell, the simulation gave us a good estimation of the temperature difference.

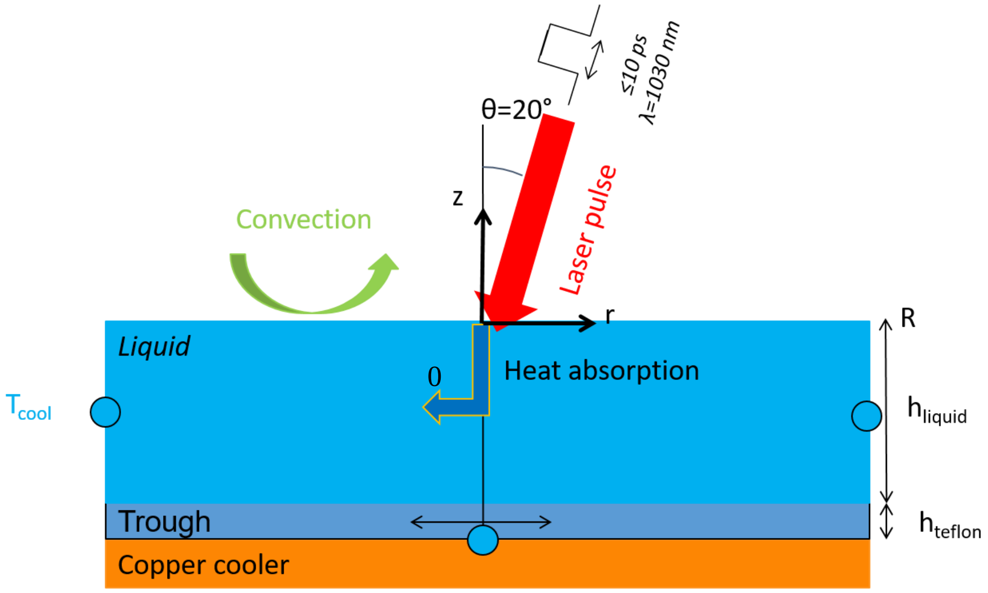

Figure S4 Schematic of the simulated system: the liquid Hg sample lying on a small Teflon cell actively cooled by a metal base plate connected to the water chiller. The heat radiation is schematized by the straight arrow in red and the convection by the curvy arrow in green.

S5.2. General equation of heat transfer

The general equation governing heat transfer in matter, at the macroscopic scale for temperature (T) is:

$$\rho(T)C_p(T)\frac{\partial T}{\partial t} + \rho(T)C_p(T)div(\vec{U}T) = div(\lambda(T)gradT) + P_v(t) \quad (10)$$

with ρ being the material density in kg·m^{-3}, C_p, the specific heat in J·kg^{-1}·K^{-1}, T, the temperature in K, $\vec{U}$, the velocity field in m·s^{-1}, λ, the heat conductivity in W·m^{-1}·K^{-1}, and P_v, the power density in W·m^{-3}.

In order to simulate this problem, the following hypotheses were assumed:

1. Although the laser is hitting the surface at an angle of 20° with respect to the vertical axis, it can still be assumed that the problem is axis-symmetric and in two dimensions (cylindrical coordinates r, z).
2. The temperature at a depth of 1mm from the laser-exposed hot surface is considered constant.

3. The copper cooler is assumed to very efficient, so that the temperature at the bottom of the trough is considered constant.
4. Due to the low height and the relatively small difference of temperature, the internal motion of the liquid can be neglected. We assume only conduction to exist in the system.
5. In the case of mercury, the high absorption coefficient allows the assumption that the non-reflected heat is absorbed totally at the surface within 12 µm depth.

S5.3. Simplified equation and boundary conditions

Considering the above hypotheses, the simplified heat transfer equation can be written as following:

$$\rho(T)C_p(T)\frac{\partial T}{\partial t} = \lambda(T)(\frac{\partial^2 T}{\partial z^2} + \frac{1}{r}\frac{\partial T}{\partial r} + \frac{\partial^2 T}{\partial r^2}) + P_v(t) \tag{11}$$

where z is the height and r is the radius (Error! Reference source not found.).

Since, in case of Hg, the power density is equal to zero, the above equation can be simplified as:

$$\lambda(T)\left(\frac{\partial^2 T}{\partial z^2} + \frac{1}{r}\frac{\partial T}{\partial r} + \frac{\partial^2 T}{\partial r^2}\right) + P_v = 0 \tag{12}$$

In the same way the boundary conditions have been set up as follows:

Surface boundary r=0 to ∞, z=0

To simulate the heat supply or removal due to the heat exchange between the air and the sample, a Fourier condition has been chosen. In the case of Hg, the heat supplied by the laser has been modelled by adding a surface power density in the Fourier condition.

$$\lambda\frac{\partial T}{\partial z} + h_{cv}(T - T_\infty) = (1 - \rho_{mercury})P_s(t) \tag{6}$$

With h_{cv} the convection coefficient in $W{\cdot}m^{-2}{\cdot}K^{-1}$, T_∞ the cell temperature in K, $P_s(t)$ the surface power density in $W{\cdot}m^{-2}$.

The convection coefficient is not calculated, thanks to correlation involving dimensionless numbers but its value is fixed at 5 $W{\cdot}m^{-2}{\cdot}K^{-1}$.

To consider the convection with air taking place at the sides of the sample trough, the below equation has been adapted.

$$\lambda\frac{\partial T}{\partial r} = -h_{cv}(T - T_\infty) \tag{13}$$

S5.4. Spatial and temporal discretization for liquid Hg sample

The solver is set up with the Backward Differentiation Formula (BDF). The temporal discretization is fixed as, from at least, 2 ps up to ten times less than the pulse width (1ps). For the spatial discretization, the build of the mesh is a very hard and tricky especially in the case of the mercury. Indeed, the bigger the instantaneous density power is, the finer the mesh must be. Moreover, a too fine mesh cannot be applied everywhere in the problem due to the lack of CPU power and space in the hardware. The following choice has been made to answer to the computing constraint while also preserving a required numerical precision. Fig. S5 shows the spatial discretization resulting of the choices previously introduced.

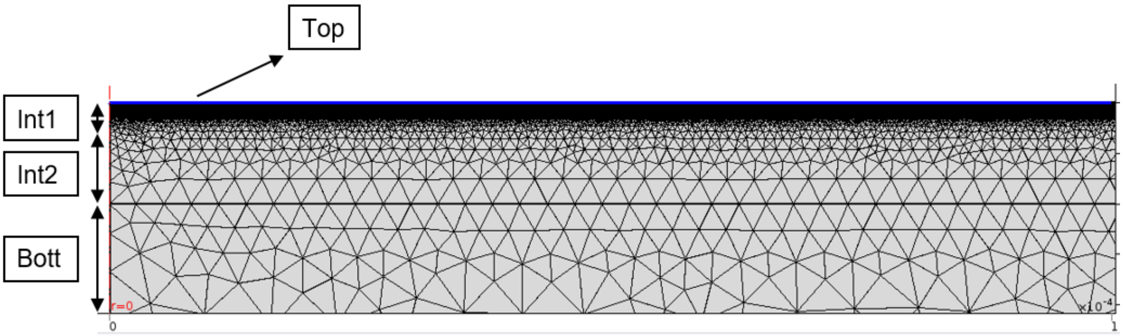

Figure S5 Mesh of the heat transfer and radiative problem. The size of the mesh top layer mesh is 3×10^{-8} m (blue line), of In Int1 3×10^{-7}m, of Int2 is 3×10^{-5} m, and of the bottom is 3×10^{-4} m. At the top the mesh is finer than as the bottom in order to provide good calculation at the fastest speed possible.

The X-ray probed volume has a maximum element size of 3.78E-8m (blue part).
Below this probed part stands an intermediate part with a maximum element size of 3.71E-7m (Int1).
Below this intermediate part, there is a part where the laser doesn't make any direct temperature change with a maximum element size of 3.71E-5m (Int2).
Finally, the trough part with a maximum element size of 3.71E-4m (Bott).

S5.5. Mercury steady state

Considering the above equations, the boundary conditions and the input parameters of our experiments, the steady state temperature after one laser pulse of width 10 ps has been calculated as a function of the sample thickness (Fig S6). The height has been considered in an interval from 0.01 mm to 10 mm.

S5.6. Geometrical parameters

Width FWMH laser: 4 mm

h_{teflon}: 0.54 mm

S5.7. Radiative parameters

Since the mercury is opaque to laser light of 1030nm wavelength, the only relevant parameter is the reflectance. It has been calculated from the refractive index and the results is 0.78 (Johnson & Christy, 1972).

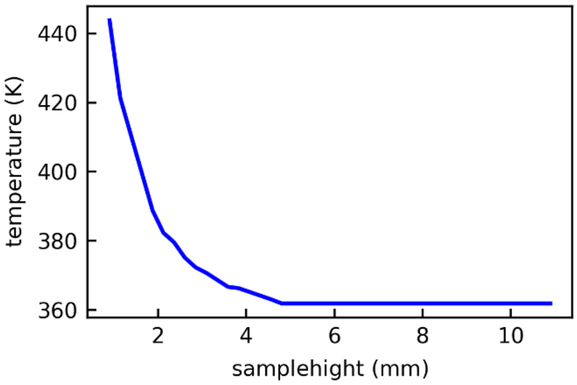

Figure S6 Steady state temperature (solids curve) for continuous power of 15W and temperature after a single of 10ps and 100µJ pulse (dashed curves) depending on the mercury height for Teflon trough (red curves) and silicon trough (blue curve). The beam diameter is 4mm and the wavelength is 1030nm.

The main finding is that the boiling point is never reached for a height of 5 mm of the sample. As a function of the height the difference of temperature is an increase of ~ 40K.

S6. Further measurements and figures

S6.1. Time resolved reflected measurements for intensity constancy checking

The Fig. S7 illustrates the measured intensity for different laser/X-ray overlap positions at the highest laser fluence of 107 µJ/cm^2. Also, for the highest possible laser fluence the measured intensity is constant during the laser exposure if the overlap between laser and X-ray is matched. For a misalignment in the spatial overlap the intensity drops during the laser exposure in dependence of the misalignment rate.

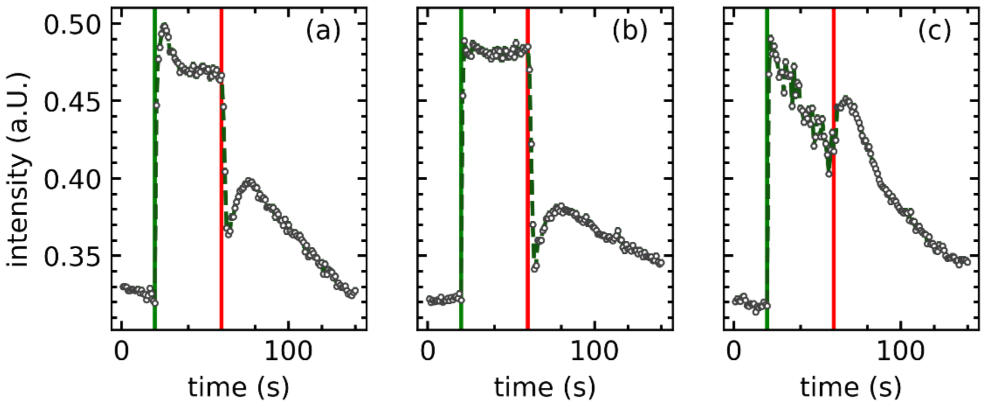

Figure S7 Specular reflected beam intensity at q_z = 0.3 $Å^{-1}$ for 3 M NaI solution at different laser/X-ray overlap positions, maxima (a), middle turning point (b) and minima (c), along the trajectory shown in Fig. 7b. The highest possible laser fluence of 107 μJ/cm^2 and a laser beam diameter of 4.0 mm^2 was used. The laser exposure starts at t = 20 s (green) and end at t = 60 s (red). In the middle turning point the reflected beam intensity keeps constant after laser exposure (green).

References

Johnson, P. B. & Christy, R. W. (1972). *Phys. Rev. B.* **6**, 4370–4379.

Weber, M. J. (2018). *Handbook of Optical Materials:* CRC Press.
